# Exploring the high-resolution mapping of gender-disaggregated development indicators

**DOI:** 10.1098/rsif.2016.0825

**Published:** 2017-04-05

**Authors:** C. Bosco, V. Alegana, T. Bird, C. Pezzulo, L. Bengtsson, A. Sorichetta, J. Steele, G. Hornby, C. Ruktanonchai, N. Ruktanonchai, E. Wetter, A. J. Tatem

**Affiliations:** 1 WorldPop, Department of Geography and Environment, University of Southampton, Southampton, UK; 2 Flowminder Foundation, Stockholm, Sweden; 3 Department of Civil and Building Engineering, Loughborough University, Loughborough, UK; 4 Stockholm School of Economics, Stockholm, Sweden; 5 Department of Public Health Sciences, Karolinska Institute, Stockholm, Sweden

**Keywords:** geo-statistics, development indicators, mapping, geographic information system

## Abstract

Improved understanding of geographical variation and inequity in health status, wealth and access to resources within countries is increasingly being recognized as central to meeting development goals. Development and health indicators assessed at national or subnational scale can often conceal important inequities, with the rural poor often least well represented. The ability to target limited resources is fundamental, especially in an international context where funding for health and development comes under pressure. This has recently prompted the exploration of the potential of spatial interpolation methods based on geolocated clusters from national household survey data for the high-resolution mapping of features such as population age structures, vaccination coverage and access to sanitation. It remains unclear, however, how predictable these different factors are across different settings, variables and between demographic groups. Here we test the accuracy of spatial interpolation methods in producing gender-disaggregated high-resolution maps of the rates of literacy, stunting and the use of modern contraceptive methods from a combination of geolocated demographic and health surveys cluster data and geospatial covariates. Bayesian geostatistical and machine learning modelling methods were tested across four low-income countries and varying gridded environmental and socio-economic covariate datasets to build 1×1 km spatial resolution maps with uncertainty estimates. Results show the potential of the approach in producing high-resolution maps of key gender-disaggregated socio-economic indicators, with explained variance through cross-validation being as high as 74–75% for female literacy in Nigeria and Kenya, and in the 50–70% range for many other variables. However, substantial variations by both country and variable were seen, with many variables showing poor mapping accuracies in the range of 2–30% explained variance using both geostatistical and machine learning approaches. The analyses offer a robust basis for the construction of timely maps with levels of detail that support geographically stratified decision-making and the monitoring of progress towards development goals. However, the great variability in results between countries and variables highlights the challenges in applying these interpolation methods universally across multiple countries, and the importance of validation and quantifying uncertainty if this is undertaken.

## 1. Introduction

The UN sustainable development goals (SDGs), an intergovernmental set of 17 aspirational goals and 169 targets to be achieved by 2030 [1], were launched in 2015. These include ending poverty and malnutrition, improving health and education, and building resilience to natural disasters and climate change. A particular focus across the goals and targets is achievement ‘everywhere’, ensuring that no one gets left behind and that progress is monitored at subnational levels to avoid national-level statistics masking local heterogeneities. This requires consistent, comparable evaluation and monitoring of key SDG indicators at high levels of subnational detail across the 2015–2030 period of the goals.

The increasing focus on subnational assessments for the SDGs, as well as for efficient targeting of resources, and the improvement in accuracy for health and development metrics has prompted an emphasis on subnational data collection and the continued development of mapping approaches. Principal among these approaches is small area estimation [2–4] whereby survey data on the variable of interest mapped at coarse spatial scales are integrated with census data at fine spatial scales to infer fine resolution mapping of key development metrics. This approach has seen widest application in the field of poverty mapping [5,6], but it is limited due to its reliance on census data. With national population censuses undertaken typically only every 10 years, and sometimes longer in many low-income countries [7], this makes the application of such small area estimation approaches challenging for the ongoing monitoring of SDG indicators.

National household surveys are undertaken in low- and middle-income countries more regularly than censuses, typically every 3–5 years, with the Demographic and Health Surveys (DHS) (http://dhsprogram.com), Living Standard Measurement Surveys (LSMS) [8,9] and Multiple Indicator Cluster Surveys (MICS) (http://mics.unicef.org) being the largest international programmes. These are vital for providing SDG, policy and operational relevant metrics; however, the data are typically only summarized at national or large subnational areas, which can be inappropriate for identifying the significant heterogeneities that need to be captured for the ‘leave no one behind’ agenda. The increasing use of global positioning systems (GPS) for recording the locations of survey clusters and the increasing availability of these data provide more fine-grained information [10]. However, these cluster-level data are drawn from small sample sizes and only represent samples of small areas. Spatial interpolation approaches that exploit spatial relationships between cluster-located survey data and geospatial covariates have, therefore, been explored recently [11,12], with applications seen in mapping age structures [13], malaria prevalence [14], vaccination coverage [15] and poverty [16] among others. Moreover, the DHS programme is now routinely providing modelled surfaces with each new country survey produced through spatial interpolation (http://spatialdata.dhsprogram.com/modeled-surfaces/) [17].

While spatial interpolation approaches are growing in popularity, due to advantages over small area estimation in their ability to produce high spatial resolution maps in the absence of census data, it remains unclear, however, how predictable different SDG-related variables are across different settings and between demographic groups. Here we test the accuracy of several spatial interpolation methods through quantification of uncertainty and model fit by combining DHS cluster data and geospatial covariates through a set of case studies, producing gender-disaggregated maps of literacy rates, stunting and the use of modern contraceptive methods. Bayesian geostatistical (BGS) and machine learning modelling methods were implemented across four low-income countries and gridded environmental and socio-economic covariate datasets to predict 1 × 1 km spatial resolution maps with uncertainty estimates, and tested through validation.

## 2. Material and methods

The study focused on three countries in sub-Saharan Africa (Nigeria, Kenya and Tanzania) and one country in south Asia (Bangladesh). Development indicators that underlie key SDGs, and for which significant gender differences often exist, were chosen for testing: literacy, stunting and the use of modern contraceptive methods.

Empirical estimates of literacy levels at national level among women of childbearing age are low in all four countries [18–21] with marked differences between men and women. For example, around 14% of Kenyan women age 15–49 cannot read at all, compared with 7% of men in the same age interval. Estimates from latest surveys show that children whose mother has no education are more than twice as likely to be short for their age (stunted) when compared with children of mothers who have completed secondary or higher education [20].

In Nigeria, Kenya and Bangladesh a relatively high proportion of children under the age of five (35–41%) are stunted. Even in Kenya where the rate of illiteracy is relatively low, the numbers of malnourished children remain high (35% of children under five are stunted with a 14% of severely stunted) [18].

In Nigeria, only 11% of the women aged 15–49 reported using a modern method of contraception [19], and just 24% in Tanzania [21].

### 2.1. Geolocated household surveys

The gender-disaggregated indicators investigated in these analyses were collected through the DHS programme, which collects and analyses data on populations through more than 300 surveys in over 90 countries. DHS household surveys adopt a multistage cluster sampling design [17–21]. Sampling clusters are usually the primary sampling units, which are pre-existing geographical areas known as census enumeration areas (EAs).

The boundaries of the EAs are defined by the country's census bureau, as are the urban and rural status of each cluster. The georeferenced datasets can be linked to individual and household records in DHS household surveys through unique cluster identifiers. To protect the confidentiality of respondents, cluster locations are displaced up to 5 km in rural areas and up to 2 km in urban areas at the processing stage. A further 1% of the rural clusters can be displaced up to 10 km [22–24]. Because displacement affects the physical location of the data, it is necessary to account for displacement when undertaking spatial modelling with DHS surveys [10].

Gender-disaggregated maps of stunting, literacy and use of modern contraceptive methods were constructed, with a subset of these indicators analysed in each country (with the exception of Nigeria, where all indicators were modelled). These indicators are clearly defined by the DHS programme and were constructed following their instructions contained in individual country final reports [18–21] as well as in [25]. Details of each are outlined below.

#### 2.1.1. Stunting in children

Indicators of nutritional status in children from DHS surveys are calculated using growth standards published by WHO [26] in 2006. Stunting is a measure of chronic malnutrition, and in some countries may be environmentally linked where the combination of poverty and low agricultural productivity limit calorific intake in children [27]. Using DHS data, children whose height-for-age *Z*-score was below minus two standard deviations (−2 s.d.) from the median of the WHO reference population are considered stunted and chronically malnourished.

A measure of stunting for children under the age of 5 who slept in the household the night before the survey was extracted for the countries of interest using height-for-age *Z*-scores. The cluster-level proportions of stunted children disaggregated by gender were used in the analyses.

#### 2.1.2. Literacy

In the DHS surveys, literacy status is determined by assessing the respondent's ability to read a sentence during the interview, when surveyors ask respondents to read sentences written in their native language or English. Those who attended at least secondary school or were able to read at least part of a sentence were defined as literate. In Bangladesh, the female literacy rate regards only ever married women. Cluster-level proportions of literate people aged 15–49 were used, disaggregated by gender.

#### 2.1.3. Use of modern contraception methods

Within the DHS surveys, all women aged between 15 and 49 years old were asked about their use of family planning at the time of the survey. Information about the current use of any modern method of contraception (defined in the DHS as being, e.g. pill, male condom and sterilization) was reported for each women interviewed. In Bangladesh, this information was only collected from ever married women. Cluster-level proportions of women using a modern method of contraception were derived and used in these analyses.

### 2.2. Defining a suite of covariates for predicting health and demographic indicators at fine spatial resolution

Many indices of population health and well-being are correlated with variables describing the surrounding environmental, geographical, socio-economic and infrastructure conditions. Spatial interpolation approaches have been developed to exploit these correlative relationships, along with the spatial autocorrelation present [13,14,28] to predict the indicators at locations where survey data are not available. Key to this approach is the availability of high-resolution geographical data that can be used to describe conditions at survey locations, as well as to predict across the rest of the area of interest.

Following previous work [13–16], a suite of physical (topography, climate, land cover, etc.) and some social (population density, ethnicity) covariate grids were selected from existing publicly available libraries and assembled, focusing on factors that have previously been shown to correlate with the modelled indicators and completely covering the selected countries (table 1). For each country, differing sets of covariate data were available, and due to the different spatial resolution, projection system, format and extent of the datasets, algorithms were developed and applied for converting all the layers to common 1 × 1 km gridded datasets suitable to be used in map production. Further information is available in the electronic supplementary material.

**Table 1.** List of geospatial covariates assembled for mapping literacy, stunting and the use of modern contraception methods.

category	covariates	data source
travel time	accessibility	European Commission Joint Research Centre (http://forobs.jrc.ec.europa.eu/products/gam)
distances	distance to settlements, roads, rivers, conflicts, schools and health facility	input data from the WorldPop Project (www.worldpop.org), Open Street map (www.openstreetmap.org), ACLED (http://www.acleddata.com/data/acled-versions-1–5-data-1997–2014/)
climate	temperature, precipitation, aridity index, potential evapotranspiration	MODIS (http://modis.gsfc.nasa.gov/), Consortium for Spatial Information (CGIAR-CSI) (www.cgiar-csi.org), WorldClim (www.worldclim.org)
satellite indices	MODIS EVI, mid-infrared index, nightlights	MODIS, NOAA VIIRS (ngdc.noaa.gov/eog)
demographic	population, births, pregnancies, ethnicity	WorldPop Project, ETH Zurich (http://www.icr.ethz.ch/data/geoepr)
topography	elevation	US Geological Survey (USGS) (http://eros.usgs.gov/elevation-products), CGIAR-CSI
environment	protected areas, percentage of urban areas	WDPA (http://protectedplanet.net/), input data from WorldPop Project
livestock densities	small ruminant, cattle, goats, pigs, poultry, sheep	FAO in collaboration with the Environmental Research Group Oxford (ERGO) (http://livestock.geo-wiki.org)
economic	gross cell product	Yale GEcon Research Project (http://gecon.yale.edu/)
land/agriculture	land cover, rainfed crop suitability	NASA/USGS (https://lpdaac.usgs.gov/dataset_-discovery/modis/modis_products_table/mcd12q1), FAO FGGD (http://geonetwork3.fao.org/fggd/), ESA Globcover (http://due.esrin.esa.int/page_globcover.php)

Owing to the displacement affecting DHS data, the mean value of each variable in a buffer of 2 km from the cluster location for urban areas and 5 km for rural areas was used, following published recommendations [10], in applying a linear modelling approach. For nonlinear modelling architectures (e.g. artificial neural networks (ANNs)), the values came from a Monte Carlo analysis on the same buffers. Further details of modelling methods are provided below.

### 2.3. Selection of geospatial covariates

Selecting an optimal set of covariates is fundamental to maximize the predictive accuracy of a model. Including too few informative covariates could result in loss of explanatory power, while the inclusion of too many could cause the resulting high-dimensional multivariate model to overfit the data, especially when an ANN is applied. In statistical modelling, selection of the better performing covariates within the chosen modelling architecture is a common, widely accepted, exercise [29]. For obtaining the most appropriate combination of covariates to predict high-resolution maps for each of the modelled indicators, a sensitivity analysis using a jackknife approach [30] was carried out.

Another challenge to modelling is multicollinearity, which can have significant impact on the quality and stability of a model. There are a number of methods for detecting multicollinearity [31]. The approach selected here was to compute the variance inflation factor (VIF)—the larger the VIF, the bigger is the multicollinearity. Multicollinearity was tested here among independent variables. Though some authors suggest excluding variables with a VIF greater than 4 [32] or 5 [33], here it was decided to safely keep only variables with a VIF lower than 3. Further information is available in the electronic supplementary material.

### 2.4. Modelling architecture

The BGS [34,35], generalized linear models [36] (simple or with mixed effect) and machine learning (ANN in this case) [37,38] techniques based on geolocated surveys and gridded spatial covariate layers were applied and tested to construct high-resolution gender-disaggregated maps of stunting, literacy and use of modern contraceptive methods. The applied modelling architecture has its basis in the geospatial semantic array programming paradigm (GeoSemAP) [39,40] with efforts focused towards computational reproducibility and semantic modularization of the many data-transformation components. A data-transformation module (D-TM) may be considered as a handy portable formulation for applying a mathematical function to a set of input data and parameters.

GeoSemAP is the geospatial application of the semantic array programming (SemAP) paradigm [41,42]. SemAP allows the multidimensional structure of a mathematical and computational model to be exploited. The modelling architecture relies on a modular structure where each D-TM is subject to semantic consistency checks in order for the input/output processed information to be compliant with the semantics underpinning the variables manipulated within the module. In an effort towards increasing reproducibility in geostatistical modelling, free scientific software tools and libraries, and freely available datasets were used, and reproducible techniques for applying the models and sub-models that are part of the modelling architecture were developed. Owing to the generally better performance we observed for BGS and ANN compared with generalized linear models in preliminary tests, all of the maps produced were based on these modelling architectures.

#### 2.4.1. Artificial neural networks

An ANN is a D-TM able to derive from a set of input data a corresponding set of outputs. Neural networks resemble the human brain because of knowledge acquisition through learning, and storage of acquired knowledge within inter-neuron connection strengths. An ANN is implemented through a system of interconnected nodes. Information propagates through nodes, transforming the inputs in intermediate derived signals up to generate the final outputs. The internal nodes are called neurons and define the ANN hidden layers. Each of the processing neurons calculates the weighted sum of all interconnected signals from the previous layer plus a bias term and then produces an output through the activation function. The effective incoming signal *s_j_* to node *j* is
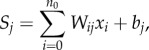
where *W_ij_* is the connection weight, *x*_*i*_ is the input to the network and *b_j_* is the bias term.

The activation function associating individual nodes typically has a sigmoid shape. The sigmoid function most often used for ANNs is the logistic function:
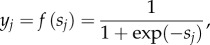
in which *s_j_* can vary in the range ±∞ but *y* is bounded between 0 and 1.

The power and main advantage of using ANNs lie in their capacity to model both linear and nonlinear relationships and to learn these relations directly from the data. Because many complex problems are characterized by their intrinsic nonlinear behaviour, traditional linear models are often inadequate.

Two different feed-forward neural networks were selected among the main network architectures. In a feed-forward network, the information moves in only one direction, from the input nodes to the output nodes without cycles or loops. The first was created using the R language according to the structure established by the ‘AMORE’ package (A MORE flexible neural network) [43] of the GNU R [44] free software. The second is a feed-forward ANN implemented in Matlab language using the Neural Network Package [45] available in GNU Octave [46]. Additional information is available in the electronic supplementary material.

The setting of the parameters for running the applied ANNs comes from a repeated random sub-sampling validation using 70% of the DHS data, keeping the remaining 30% for the final validation of the models.

#### 2.4.2. Bayesian generalized linear models

A Bayesian modelling approach is a statistical technique that uses the Bayesian method [47,48] to estimate the parameters of the posterior distribution. Because it incorporates a hierarchical analysis in the observation data model, prior distributions and data likelihood with associated uncertainty for parameters specified [48], the Bayesian approach is a valid contribution for modelling large datasets that also include spatial information. Bayesian predictions are accompanied by measures of uncertainty and these have been used in many applications focused on the spatial modelling of development and health indicators [14,49].

The integrated nested Laplace approximations (INLA) approach, available as package implemented in GNU R, was applied here [50]. It is a powerful and computationally effective alternative to classic simulation methods such as the Markov chain Monte Carlo, that can become computationally intensive for large datasets. This is an approach to statistical inference for latent Gaussian Markov random field models as described in [50]. Latent Gaussian models are a wide, flexible class of models that include (generalized) linear, mixed, spatial and spatio-temporal models. Combined with the stochastic partial differential equation approach (SPDE) [51], it is possible to model all kinds of geographically referenced data.

To produce continuous maps of the estimated proportion of gender-disaggregated literacy, stunting and use of modern contraception methods, Bayesian hierarchical spatial models, implemented through a SPDE approach, were created using the R INLA package.

A summarized form of these models can be represented as
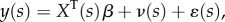
where *y*(*s*) was the realization of the overall process at the cluster location *s_i_*, *i* = 1, … , *n*. The mean structure *μ*(*s*) = *X*^T^(*s*)*β* is driven by the covariates. The residual structure is then partitioned into *v*(*s*) arising from zero-centred stationary Gaussian process capturing the spatial association at cluster level and the *e*(*s*) ∼ *N*(0, *σ_e_*) as the uncorrelated error terms. This spatial association is implemented through a solution to the SPDE expressed as
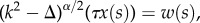
where (*k*^2^ − Δ)*^α^*^/2^ is a differential operator, *k* is a scaling parameter, Δ is the Laplacian, *α* controls the smoothness of realization, *τ* controls the variance and *x*(*s*) is the spatial field/domain for *s* (*s*_1_, … , *s_n_*) locations.

Because of the time necessary to properly calibrate the applied models, after an initial test where the results of many different modelling architectures were compared, the decision was made to initially apply only the models implemented in INLA. These models are considerably less time demanding than ANNs but have a similar predictive capacity (table 2). ANNs were additionally tested against the BGS models only when poor results in prediction were obtained and, in particular, to overcome the problem of unusual data distributions.

**Table 2.** Comparison of five different models (logistic regression (LR), ANN and BGS) for calculating the proportions of literate ever married women in Bangladesh based on validation statistics (mean square error (MSE), explained variance).

model	MSE (valid)	exp. var. (valid)
INLA	0.025	0.18
INLA (SPDE)	0.023	0.24
LR	0.025	0.19
ANN (R)	0.023	0.24
ANN (Octave)	0.022	0.27

### 2.5. Model validation

Both the ANN models and those implemented in INLA use a cross-validation (repeated random sub-sampling) to set the model parameters. Owing to the size of the Nigeria datasets, here we split the data into training, validation and test sets, using the validation dataset (20% of the data) for building the final model.

The validation process was implemented in two steps. First, the cross-validation approach (with the exception of Nigeria) was applied to the training dataset for selecting the best model for each of the applied modelling architectures (ANN, BGS). The relationship between predicted and observed values (the accuracy of the model) was quantified using the root mean square error (RMSE) and the mean absolute error (MAE). Although some authors suggest inter-comparisons of average model performance should be based on MAE [52], RMSE was also calculated here because of its greater sensitivity to occasional large error compared to other measures. The remaining 30% of the data (20% in Nigeria) were used for measuring the modelling performance by calculating MAE, RMSE and the explained variance of the model (expressed in proportional terms).

The model with the highest explained variance and lowest RMSE and MAE was selected to be applied for producing the final map at 1 × 1 km resolution. For calculating the explained variance the pseudo-*R*^2^ reported in equation (2.1) was used:2.1
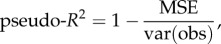
where var(obs) is the variance of the observed data and MSE is the mean square error.

A comparison of RMSE and MAE of different models based on different datasets may only capture part of the relevant statistical information. For example, both RMSE and MAE indices cannot directly preserve the information concerning the sign of the modelling errors. In particular, a model with given RMSE and MAE may locally display errors both negative (underestimation) and positive (overestimation) so as for the overall bias to be mitigated by the compensating local under/over-estimations. Another model with the same RMSE and MAE may instead systematically underestimate the modelled quantity. In order for these modelling situations to be better discriminated, we introduced a new parameter for calculating the general bias of the models (equation (2.2)):2.2
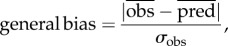
where *σ*_obs_ and 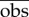 are the standard deviation and mean of observed data and 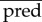 is the mean of predicted values.

## 3. Results

The following sections document the performance of the applied modelling architectures in each of the investigated countries. Example maps and graphs for selected indicators are presented, with further results provided in the electronic supplementary material information. The results highlight that relatively accurate high-resolution maps of key gender-disaggregated socio-economic indicators can be produced, with explained variance through validation being as high as 74–75% for female literacy in Nigeria and Kenya, and in the 50–70% range for many other variables. However, substantial variations between countries and variables were seen, with many variables showing poor mapping accuracies in the range of 2–30% explained variance using both geostatistical and machine learning approaches.

Both Bayesian modelling techniques and ANNs were able to extract the information present within the available covariates to predict development indicators in different areas. Mid-infrared reflectance (the surface reflectance in the middle infrared part of the electromagnetic spectrum), elevation, accessibility, the distance to settlements and roads and the distance to conflicts (Nigeria) emerged as important covariates for mapping different indicators. For example, the mid-infrared reflectance shows a correlation of 0.58, 0.63 and 0.7 with stunting of females in Nigeria and female literacy in Kenya and Nigeria, respectively. However, not all countries and not all modelling outputs produced such a high correlation between the dependent variable and available covariates. Modelling the proportion of stunted girls under the age of 5 in Kenya, the distance to roads was found to have the highest correlation, but this was only 0.13.

### 3.1. Nigeria

**Figure 1.**
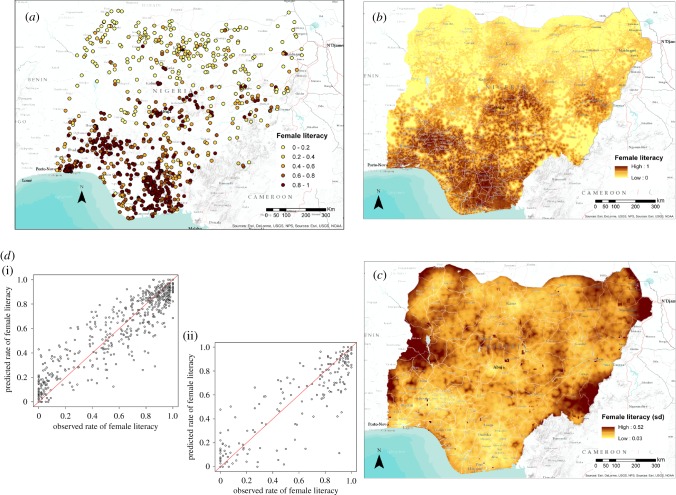
(*a*) The distribution of cluster-level data from the DHS household survey in Nigeria showing the proportion of women aged 15–49 that were classified as literate. (*b*,*c*) Map of the mean predicted proportion of literacy in Nigeria for women age 15–49 at 1 km^2^ resolution (*b*) and related uncertainty map (*c*) showing its standard deviation. (*d*) Scatter plot of the estimated proportions of female literacy in Nigeria (*y*-axis) by observed data (*x*-axis) for the training (i) and validation (ii) subset of data. (Online version in colour.)

**Figure 2.**
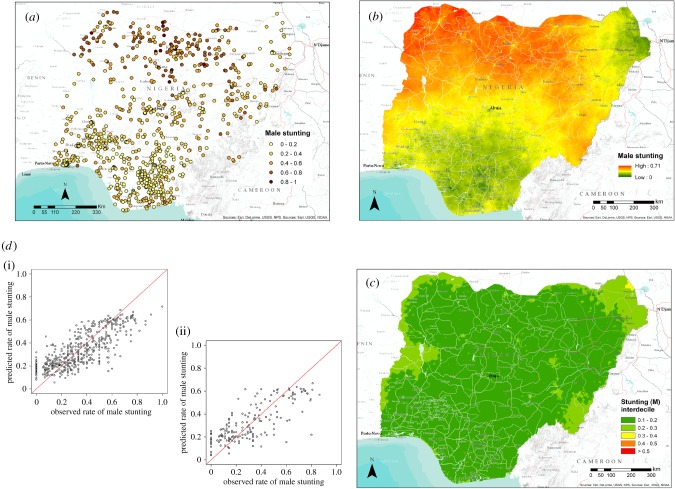
(*a*) The distribution of cluster-level data from the DHS household survey in Nigeria showing the proportion of male children under age 5 that were classified as stunted. (*b*,*c*) Map of the mean predicted proportion of stunting at 1 km^2^ resolution (*b*) and related uncertainty map (*c*) showing its interdecile range. (*d*) Scatter plot of the predicted proportion of stunted male children (*y*-axis) by observed data (*x*-axis) for the training (i) and validation (ii) subset of data. (Online version in colour.)

Table 3 shows the statistics produced for all Nigeria models, with consistently high levels of explained variance—all with a proportion above 0.57 and as high as 0.74 for female literacy. Figures 1 and 2 show input data, scatterplots and output maps for female literacy and stunting in boys, with substantial geographical heterogeneity present and relatively consistent and low levels of uncertainty in the predictions across the country. For example, in the female literacy map more than 73% of the pixels have a standard deviation lower than 0.1 and less than 0.1% of the cells show a value higher than 0.2.

**Table 3.** Modelling results related to different gender-disaggregated development indicators in Nigeria. RMSE, MAE, explained variance, MSE and MSE of a trivial model (mean) were calculated.

country	modelled parameter	modelling technique	MSE	RMSE	MAE	exp. var.	MSE (mean)
Nigeria	female literacy	INLA	0.03	0.18	0.133	0.74	0.12
Nigeria	male literacy	INLA	0.04	0.20	0.145	0.57	0.096
Nigeria	female stunting	INLA	0.020	0.143	0.112	0.61	0.052
Nigeria	male stunting	INLA	0.021	0.146	0.117	0.60	0.053
Nigeria	modern cont. met.	INLA	0.005	0.073	0.057	0.58	0.012

Key covariates across the different variables modelled were the distance from conflicts, the mid-infrared reflectance values, the gross cell product and elevation.

### 3.2. Kenya

**Figure 3.**
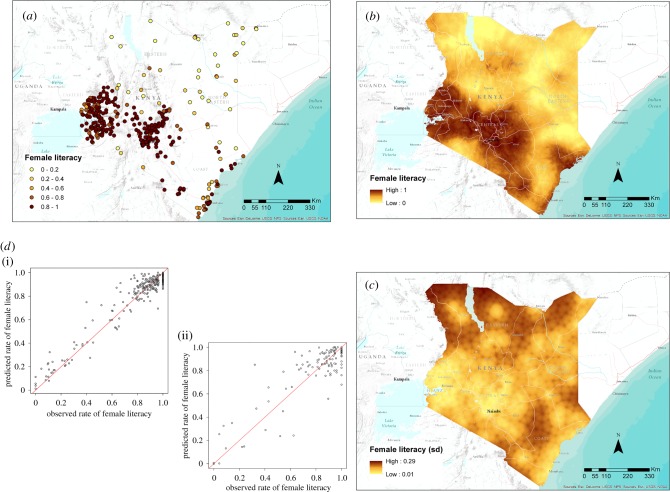
(*a*) The distribution of cluster-level data from the DHS household survey in Kenya showing the proportion of women aged 15–49 that were classified as literate. (*b*,*c*) Map of the mean predicted proportion of female literacy at 1 km^2^ resolution (*b*) and related uncertainty map (*c*) showing its standard deviation. (*d*) Scatter plot of the predicted proportion of female literacy (*y*-axis) by observed data (*x*-axis) for the training (i) and validation (ii) subset of data. (Online version in colour.)

Table 4 presents the statistics related to all the models applied in Kenya. The results are heterogeneous with high levels of explained variance in modelling female literacy (0.75) and low levels for all other indicators. Figure 3 shows input data, scatterplots and output maps for female literacy. The related standard deviation is always lower than 0.3 with more than 80% of the pixels having a value of less than 0.2. Key covariates across the different variables modelled were the distance from settlements and roads, the accessibility, the mid-infrared index values and the potential evapotranspiration.

**Table 4.** Modelling results related to different gender-disaggregated development indicators in Kenya. RMSE, MAE, explained variance, MSE and MSE of a trivial model (mean) were calculated.

country	modelled parameter	modelling technique	MSE	RMSE	MAE	exp. var.	MSE (mean)
Kenya	female literacy	INLA	0.016	0.127	0.09	0.75	0.065
Kenya	male literacy	INLA	0.021	0.144	0.10	0.32	0.030
Kenya	female stunting	INLA	0.054	0.23	0.186	0.04	0.056
Kenya	female stunting	ANN (Octave)	0.054	0.23	0.186	0.04	0.056
Kenya	male stunting	INLA	0.062	0.25	0.20	0.02	0.0628

### 3.3. Tanzania

**Figure 4.**
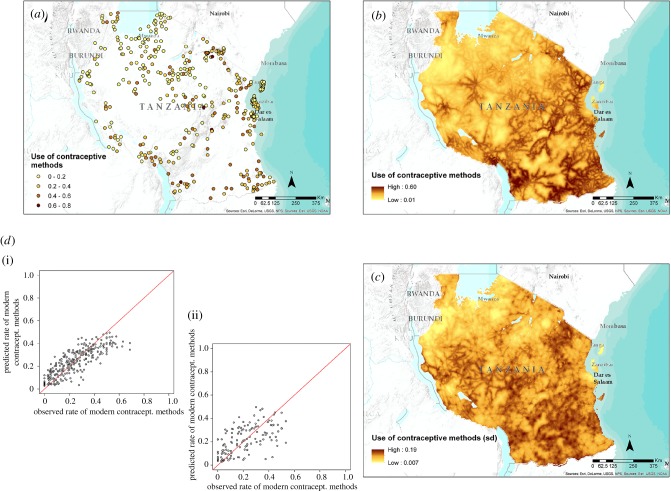
(*a*) The distribution of cluster-level data from the DHS household survey in Tanzania showing the proportion of women aged 15–49 using modern contraceptive methods. (*b*,*c*) Map of the mean predicted proportion of women using modern contraceptive methods at 1 km^2^ resolution (*b*) and related uncertainty map (*c*) showing its standard deviation. (*d*) Scatter plot of the predicted proportion of women using modern contraceptive methods (*y*-axis) by observed data (*x*-axis) for the training (i) and validation (ii) subset of data. (Online version in colour.)

Table 5 shows the statistics produced for all the models applied in Tanzania, with a medium-to-low proportion of explained variance ranging from 0.1 to 0.42. Figure 4 shows input data, scatterplots and output maps related to the use of modern contraception methods in women, with low levels of uncertainty in the predictions across the country. Key covariates across the different variables modelled were the distance from roads, accessibility, aridity index and precipitation.

**Table 5.** Comparison of different modelling results related to gender-disaggregated development indicators in Tanzania. RMSE, MAE, explained variance, MSE and MSE of a trivial model (mean) were calculated.

country	modelled parameter	modelling technique	MSE	RMSE	MAE	exp. var.	MSE (mean)
Tanzania	female literacy	INLA	0.023	0.15	0.1159	0.42	0.040
Tanzania	male literacy	INLA	0.045	0.21	0.16	0.08	0.050
Tanzania	male literacy	ANN (R)	0.044	0.21	0.15	0.10	0.050
Tanzania	modern cont. met.	INLA	0.015	0.12	0.096	0.35	0.024
Tanzania	modern cont. met.	ANN (R)	0.0157	0.125	0.10	0.33	0.024

### 3.4. Bangladesh

**Figure 5.**
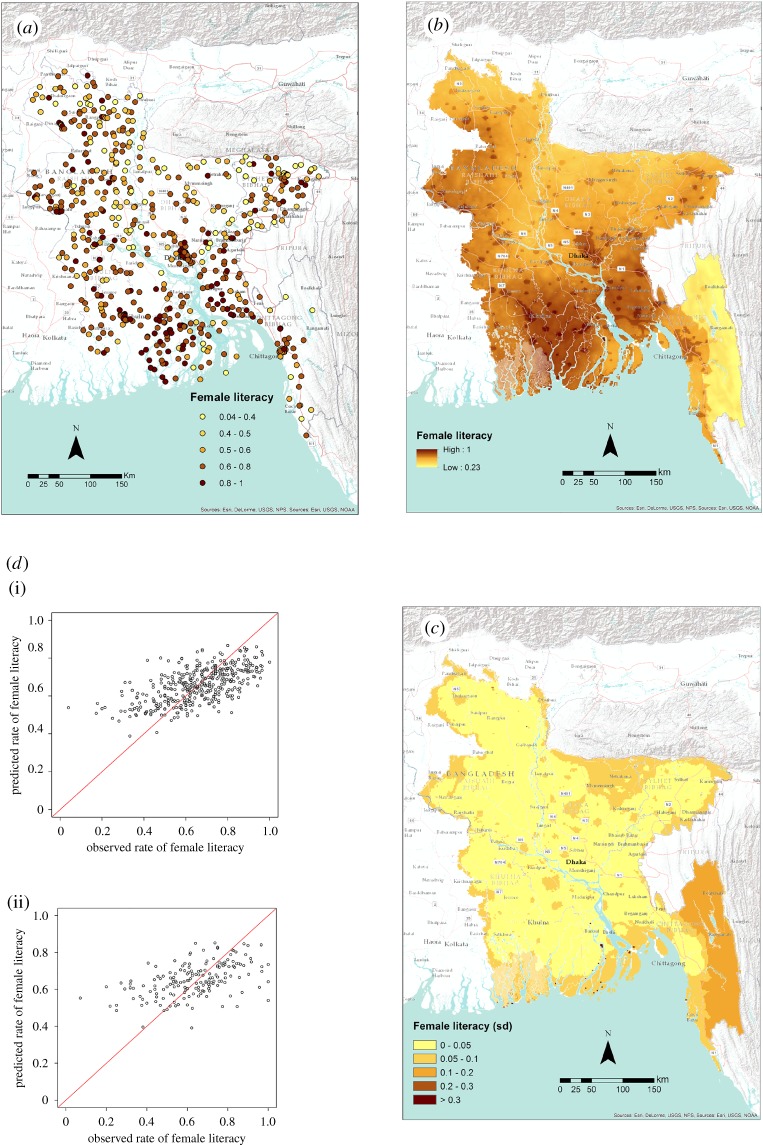
(*a*) The distribution of cluster-level data from the DHS household survey in Bangladesh showing the proportion of women aged 15–49 that were classified as literate. (*b*,*c*) Map of the mean predicted proportion of female literacy at 1 km^2^ resolution (*b*) and related uncertainty (*c*) showing its standard deviation. (*d*) Scatter plot of the predicted proportion of female literacy (*y*-axis) by observed data (*x*-axis) for the training (i) and validation (ii) subset of data. (Online version in colour.)

Table 6 shows the statistics related to all the models applied in Bangladesh. The level of variance explained by the models is low, with some of the models having performance similar to a trivial model based on the mean of the data. Figure 5 shows input data, scatterplots and output maps related to female literacy. Some of the key covariates across the different variables modelled were the distance from waterways, and the accessibility and urbanization of the area.

**Table 6.** Comparison of neural networks and Bayesian models for different gender-disaggregated development indicators in Bangladesh. RMSE, MAE, explained variance, MSE and MSE of a trivial model (mean) were calculated.

country	modelled parameter	modelling technique	MSE	RMSE	MAE	exp. var.	MSE (mean)
Bangladesh	female literacy	ANN (Octave)	0.022	0.15	0.12	0.27	0.032
Bangladesh	female literacy	INLA	0.024	0.15	0.12	0.24	0.032
Bangladesh	male literacy	INLA	0.056	0.24	0.19	0.11	0.064
Bangladesh	female stunting	ANN (R)	0.061	0.25	0.2	0.04	0.064
Bangladesh	female stunting	INLA	0.060	0.246	0.20	0.04	0.064
Bangladesh	male stunting	INLA	0.048	0.22	0.17	0.02	0.049

## 4. Discussion

The focus of the SDGs on reaching the furthest behind first creates a need for approaches that can identify who and where these people are to be able to reach them. Moreover, regular updates to such information are required to be able to track progress towards meeting the goals. Traditional data sources, such as census data on their own or integrated with survey data, can provide detailed data on some indicators for specific snapshots in time, but are limited by the irregularity of population censuses. Here we have explored the potential of spatial interpolation methods built on geolocated survey data, which are growing in popularity and application [13–17] to meet these needs through a series of case studies. The results show the potential of the approaches, and also reveal challenges in constructing consistently accurate layers across regions and variables.

The results highlight clearly that producing high-resolution maps of development indicators using spatial interpolation approaches is a challenge in some cases. There are many obstacles, starting from the lack of input data and the difficulty in selecting and setting the most appropriate modelling architecture, to the difficulties in selecting the right modelling proxy. Here we modelled 16 different indicators in four countries. In six of the 16 maps we produced, the value of the variance explained by the model was around 0.6 or higher (tables 3–6), but other models did not perform well, with values of explained variance around 0.1 or lower.

In some countries very different results were produced in modelling the same gender-disaggregated indicator. In modelling literacy (figures 1, 3 and 5), the predictive capacity of our models was lower for males than for females (tables 3–6). For Nigeria, the proportion of variance explained by the model for male literacy was satisfactory (0.57), but in Kenya even the best of the models explained only 0.32 of the variance, whereas 0.75 was achieved for female literacy. Comparison of the results of ANN and BGS when used to model the same indicators shows they have similar performance. The ANN results were better for modelling female literacy in Bangladesh, and male literacy in Tanzania, but in modelling modern use of contraception methods in Tanzania, the Bayesian architecture performed better (tables 5 and 6). If the distribution of the modelled variable is substantially different from Gaussian, the ANN approach generally appears to perform slightly better than BGS.

A number of factors underlie the differences seen in modelling performance between countries and variables, principally driven by the amount and spatial scale of variation displayed by each indicator, and the extent to which the indictor was associated with and/or driven by the available geospatial covariates. If limited or no information is present within the covariates, the models unsurprisingly fail to predict the phenomena well, but good performance was obtained where strong correlations existed. Some covariates showed strong correlations with the development indicators being modelled. For example, the correlation of travel times with literacy in Kenya was between 0.5 and 0.6 for males and females; however, this was only between 0.2 and 0.27 in Nigeria. By contrast, middle infrared reflectance in Nigeria showed a high correlation with both female stunting and literacy, but showed a correlation of only 0.1 for stunting in girls in Kenya. In some cases, therefore, the covariates likely did not relate to the driving factors behind the spatial heterogeneity seen, and additional geospatial covariates that encompass factors relating to socio-economic differences are required to capture these country-specific sociological dynamics in the models.

Precipitation, temperature and vegetation cover [53,54] have been found to be important correlates of malnutrition. Temperature, for example, is directly linked to aridity, which in turn has an impact on malnutrition [54], and the enhanced vegetation index (EVI) was found to be a significant common factor in describing rates of stunting [54]. Other examples of biophysical and geographical factors often cited in the literature include evapotranspiration, productivity of agricultural lands, distance to urban areas, topography and access to markets through road networks [55,56]. Unfortunately, the literature on this subject remains sparse because survey data rarely include metrics of these factors [56].

Correlations between literacy and urbanization have been shown previously [57], and use of contraception methods is also known to be strongly associated with levels of education, socio-economic status and access to health facilities [58,59]. In some previous work [60], it has been also shown that there exists a possible correlation between road networks and literacy, which is likely related to ease of access to schools and market. Other covariates have been tested to explore their possible indirect links with the selected health and development indicators. For example, middle infrared reflectance has previously been used to exploit its link with vegetation (vegetation spectral signatures are characterized by low reflectance in middle infrared). Nevertheless, our study shows that the same covariates can have different behaviours in different countries and occasionally also within the same country. For example, literacy rates in Nigeria have a relatively high correlation with urbanization (0.53 and 0.42 for female and male literacy, respectively), whereas female literacy rate in Kenya, despite good modelling performance, shows urbanization and literacy to be poorly correlated (0.17). Many reasons are likely behind this and further studies are necessary to better understand these differences.

Even when model performance was satisfactory here, some sources of errors contributed to the uncertainty of the model. The introduction of cluster location random displacement for protecting the anonymity of the respondent population can introduce further uncertainty to the modelled relationships [12,22]. This potential error was mitigated by extracting mean values through a defined buffer around the survey points [10]. The extent of the impact of the displacement can vary between indicators and different survey datasets but, in general, its impact on modelling performance should be modest [12]. In addition to the displacement issues, in urban areas the covariates used do not capture well the local spatial scales of variation. In general, urban areas were predicted with the same homogeneous values, not capturing any intra-city variation. Upcoming datasets (e.g. the global human settlement layer [61] and global urban footprint [62]) could lead to a future better representation of within-city variation [12].

The work presented should be considered as a preliminary study to test the strength and limits of spatial interpolation approaches. Future work should, therefore, focus on refinements of methods. This may, for example, include updating accessibility layers to include more recent and detailed road networks and settlement layers. Moreover, it could also involve modelling key driving factors of the phenomena under study, such as poverty or access to sanitation, and then using these as covariates themselves. The effect that a country-specific focus, tailored as much as possible to a specific indicator, can have on mapping accuracies rather than using globally consistent covariates should be explored. In addition, many socio-economic factors, not captured by the suite of covariates we used, and often available at aggregate levels such as administrative units, could be obtained and their ability to improve mapping accuracy tested. A challenging, but potentially very fruitful next step, could also come from integrating community based household surveys (e.g. DHS), data from governmental monitoring systems and data from different civic systems (schools, health facilities) and comparing different predictive surfaces.

The rising international focus on inequalities in the SDG era requires a detailed and strong evidence base with an explicit quantification of uncertainties. Some of the maps produced in this study have a sufficiently accurate prediction capacity to be summarized to a level of administrative unit that is relevant for policy-making and the allocation of resources. In particular, the maps of female literacy in Nigeria and Kenya, use of modern contraception methods in Nigeria or male and female stunting in Nigeria have reasonable levels of accuracy to be used for planning purposes.

The work undertaken here shows the value of combining data from geolocated household surveys with spatial covariates within advanced modelling architectures, and such approaches are growing in popularity and impact [13–16] with provision of surfaces now being a regular output accompanying new surveys [17]. However, limitations and warnings about extending such approaches across varying geographies and indicators are clear. The variability in model performance between countries and variables highlights the need for tailored approaches and robust methods with full quantification of model uncertainty to communicate where poor model fits exist. With geolocated household surveys being undertaken regularly, the potential exists for the continuous update and monitoring of SDG-relevant indicators across wide areas, but results here highlight that caution is needed.

## Supplementary Material

Supplementary Information

## References

[1] United Nations. 2016 The sustainable development goals report 2016, p. 56. New York, NY: United Nations Publications.

[2] Ghosh M, Rao JNK 1994 Small area estimation: an appraisal. Stat. Sci. 9, 55–76. (10.1214/ss/1177010647)

[3] Rao JNK 1999 Some recent advances in model-based small area estimation. Surv. Methodol. 25, 175–186.

[4] Rao JNK, Molina I 2015 Small area estimation, 2nd edn New York, NY: Wiley.

[5] Elbers C, Lanjouw JO, Lanjouw P 2002 Micro-level estimation of welfare. World Bank Policy Research Working Paper, no. 2911. Washington, DC: World Bank.

[6] Elbers C, Lanjouw JO, Lanjouw P 2003 Micro-level estimation of poverty and inequality. Econometrica 71, 355–364. (10.1111/1468-0262.00399)

[7] Tatem AJ et al. 2012 Mapping populations at risk: improving spatial demographic data for infectious disease modeling and metric derivation. Popul. Health Metr. 10, 8. (10.1186/1478-7954-10-8) PMC3487779 22591595

[8] Grosh ME, Glewwe P 1995 A guide to living standards measurement study surveys and their data sets. Washington, DC: World Bank Publications.

[9] World Bank. 2016 LSMS—living standards measurement study. See http://econ.worldbank.org/WBSITE/EXTERNAL/EXTDEC/EXTRESEARCH/EXTLSMS/0,,menuPK:3359053~pagePK:64168427~piPK:64168435~theSitePK:3358997,00.html.

[10] Perez-Haydrich C, Warren JL, Burgert CR, Emch ME 2013 Guidelines on the use of DHS GPS data. Spatial Analysis Reports, no. 8. Calverton, MD: ICF International.

[11] Burgert CR 2014 Spatial interpolation with demographic and health survey data: key considerations. DHS Spatial Analysis Reports, no. 9. Rockville, MD: ICF International.

[12] Gething P, Tatem A, Bird T, Burgert-Brucker CR 2015 Creating spatial interpolation surfaces with DHS data. Spatial Analysis Reports, no. 11. Rockville, MD: ICF International.

[13] Alegana VA, Atkinson PM, Pezzulo C, Sorichetta A, Weiss D, Bird T, Erbach-Schoenberg E, Tatem AJ 2015 Fine resolution mapping of population age-structures for health and development applications. J. R. Soc. Interface 12, 20150073 (10.1098/rsif.2015.0073) 25788540 PMC4387535

[14] Bhatt S et al. 2015 The effect of malaria control on *Plasmodium falciparum* in Africa between 2000 and 2015. Nature 526, 207–211. (10.1038/nature15535) 26375008 PMC4820050

[15] Takahashi S, Metcalf CJE, Ferrari MJ, Moss WJ, Truelove SA, Tatem AJ, Grenfell BT, Lessler J 2015 Reduced vaccination and the risk of measles and other childhood infections post-Ebola. Science 347, 1240–1242. (10.1126/science.aaa3438) 25766232 PMC4691345

[16] Tatem AJ, Gething P, Pezzulo C, Weiss D, Bhatt S 2014 Development of high-resolution gridded poverty surfaces. WorldPop report. http://www.worldpop.org.uk/resources/docs/Poverty-mapping-report.pdf.

[17] Burgert-Brucker CR, Dontamsetti T, Mashall A, Gething P 2016 Guidance for use of the DHS program modeled map surfaces. DHS Spatial Analysis Reports, no. 14. Rockville, MD: ICF International.

[18] Kenya National Bureau of Statistics (Knbs) & MEASURE DHS, ICF Macro. 2010 Kenya Demographic and Health Survey 2008–09. Calverton, MD: KNBS and ICF Macro.

[19] National Population Commission (NPC) & ICF International. 2014 Nigeria demographic and health survey 2013, p. 566. Abuja, Nigeria and Rockville, MD: NPC and ICF International.

[20] National Institute of Population Research and Training (NIPORT), Mitra and Associates & ICF International. 2013 Bangladesh Demographic and Health Survey 2011, p. 458. Dhaka, Bangladesh and Calverton, MD: NIPORT, Mitra and Associates and ICF International.

[21] National Bureau of Statistics (NBS) & ICF Macro. 2011 Tanzania Demographic and Health Survey 2010, p. 482. Dar es Salaam, Tanzania: NBS and ICF Macro.

[22] Burgert CR, Colston J, Roy T, Zachary B 2013 Geographic displacement procedure and georeferenced data release policy for the demographic and health surveys. DHS Spatial Analysis Reports, no. 7. Calverton, MD: ICF International.

[23] Macro International Inc. 1996 Sampling manual. Demographic and health surveys phase III. DHS III basic documentation, no.6. Calverton, MD: Macro International.

[24] ICF International. 2012 Demographic and health survey sampling and household listing manual. Calverton, MD: ICF International.

[25] Rutstein S, Rojas G 2003 Guide to DHS statistics: demographic and health surveys methodology. Calverton, MD: ORC Macro.

[26] WHO Multicentre Growth Reference Study Group. 2006 WHO Child Growth Standards: methods and development, p. 312. Geneva, Switzerland: World Health Organization.

[27] Giroux SC 2008 Child stunting across schooling and fertility transition: evidence from sub-Saharan Africa. DHS Working Papers, no. 57. Calverton, MD: Macro International.

[28] Sedda L, Tatem AJ, Morley DW, Atkinson PM, Wardrop NA, Pezzulo C, Sorichetta A, Kuleszo J, Rogers DJ 2015 Poverty, health and satellite-derived vegetation indices: their inter-spatial relationship in West Africa. Int. Health 7, 99–106. (10.1093/inthealth/ihv005) 25733559 PMC4357798

[29] Murtaugh PA 2009 Performance of several variable-selection methods applied to real ecological data. Ecol. Lett. 12, 1061–1068. (10.1111/j.1461-0248.2009.01361.x) 19702634

[30] Tukey JW 1958 Bias and confidence in not quite large samples. Ann. Math. Stat. 29, 614–623. (10.1214/aoms/1177706647)

[31] Ismartini P, Sunaryo S, Setiawan S 2010 The jackknife interval estimation of parameters in partial least squares regression model for poverty data analysis. IPTEK J. Technol. Sci. 21, 118–123. (10.12962/j20882033.v21i3.42)

[32] Pan Y, Jackson RT 2008 Ethnic difference in the relationship between acute inflammation and serum ferritin in US adult males. Epidemiol. Infect. 136, 421–431. (10.1017/S095026880700831X) 17376255 PMC2870810

[33] Rogerson P 2001 Statistical methods for geography. London, UK: SAGE.

[34] Blangiardo M, Cameletti M 2015 Spatial and spatio-temporal Bayesian models with R-INLA. Chichester, UK: John Wiley and Sons, Inc.

[35] Congdon P 2002 Bayesian statistical modelling. Meas. Sci. Technol. 13, 643 (10.1088/0957-0233/13/4/703)

[36] McCullagh P, Nelder JA 1989 Generalized linear models, 2nd edn Boca Raton, FL: Chapman and Hall/CRC.

[37] Michalski RS, Carbonell JG, Mitchell TM 2013 Machine learning: an artificial intelligence approach. Berlin, Germany: Springer.

[38] Yegnanarayana B 2009 Artificial neural networks. New Delhi, India: PHI Learning Pvt. Ltd.

[39] Bosco C, Sander G 2015 Estimating the effects of water-induced shallow landslides on soil erosion. IEEE Earthzine 7, 910137 (10.1101/011965)

[40] Caudullo G 2014 Applying geospatial semantic array programming for a reproducible set of bioclimatic indices in Europe. IEEE Earthzine 7, 877975 (10.1101/009589)

[41] de Rigo D 2012 Semantic array programming with Mastrave—introduction to semantic computational modelling. See http://mastrave.org/doc/MTV-1.012-1.htm

[42] de Rigo D 2012 Semantic array programming for environmental modelling: application of the Mastrave library. In *Int. Environ. Model. Softw. Soc. IEMSs 2012 Int. Congr. Environ. Model. Softw. Manag. Resour. Ltd. Planet Pathw. Vis. Uncertain. Sixth Bienn. Meet.*, pp. 1167–1176.

[43] Castejón Limas M, Ordieres Meré JB, González Marcos A, Martínez de Pisón Ascacibar FJ, Pernía Espinoza AV 2014 A MORE flexible neural network package. 10.1016/j.neunet.2004.11.007 15795116

[44] R Development Core Team. 2014 R: a language and environment for statistical computing.Vienna, Austria: R Development Core Team.

[45] Schmid MD 2009 A neural network package for Octave. User's guide version: 0.1.9.1.

[46] Eaton JW, Bateman D, Hauberg S 2009 Gnu octave version 3.0.1 manual: a high-level interactive language for numerical computations, 1st edn New York, NY: CreateSpace Independent Publishing Platform.

[47] Press SJ (ed) 2002 Hierarchical Bayesian modeling, pp. 336–358. Hoboken, NJ: John Wiley & Sons, Inc.

[48] Gelman A, Hill J 2007 Data analysis using regression and multilevel/hierarchical models, 1st edn Cambridge, UK: Cambridge University Press.

[49] Alegana VA et al. 2016 Advances in mapping malaria for elimination: fine resolution modelling of *Plasmodium falciparum* incidence. Sci. Rep. 6, 29628 (10.1038/srep29628) 27405532 PMC4942778

[50] Rue H, Martino S, Chopin N 2009 Approximate Bayesian inference for latent Gaussian models by using integrated nested Laplace approximations. J. R. Stat. Soc. B Stat. Methodol. 71, 319–392. (10.1111/j.1467-9868.2008.00700.x)

[51] Lindgren F, Rue H, Lindström J 2011 An explicit link between Gaussian fields and Gaussian Markov random fields: the stochastic partial differential equation approach. J. R. Stat. Soc. B Stat. Methodol. 73, 423–498. (10.1111/j.1467-9868.2011.00777.x)

[52] Willmott C, Matsuura K 2005 Advantages of the mean absolute error (MAE) over the root mean square error (RMSE) in assessing average model performance. Clim. Res. 30, 79–82. (10.3354/cr030079)

[53] Grace K, Davenport F, Funk C, Lerner AM 2012 Child malnutrition and climate in sub-Saharan Africa: an analysis of recent trends in Kenya. Appl. Geogr. 35, 405–413. (10.1016/j.apgeog.2012.06.017)

[54] Kinyoki DK, Kandala N-B, Manda SO, Krainski ET, Fuglstad G-A, Moloney GM, Berkley JA, Noor AM 2016 Assessing comorbidity and correlates of wasting and stunting among children in Somalia using cross-sectional household surveys: 2007 to 2010. BMJ Open 6, e009854 (10.1136/bmjopen-2015-009854) PMC4785320 26962034

[55] Balk D, Storeygard A, Levy M, Gaskell J, Sharma M, Flor R 2005 Child hunger in the developing world: an analysis of environmental and social correlates. Food Policy 30, 584–611. (10.1016/j.foodpol.2005.10.007)

[56] de Sherbinin A 2011 The biophysical and geographical correlates of child malnutrition in Africa. Popul. Space Place 17, 27–46. (10.1002/psp.599)

[57] Arouri MEH, Youssef AB, Nguyen-Viet C, Soucat A 2014 Effects of urbanization on economic growth and human capital formation in Africa. PGDA Working Paper, no. 119. Cambridge, MA: Harvard University.

[58] Stephenson R, Baschieri A, Clements S, Hennink M, Madise N 2007 Contextual influences on modern contraceptive use in sub-Saharan Africa. Am. J. Public Health 97, 1233–1240. (10.2105/AJPH.2005.071522) 17538071 PMC1913073

[59] Tuoane M, Diamond I, Madise N 2003 Use of family planning in Lesotho: the importance of quality of care and access. Afr. Popul. Stud. 18, 105–132.

[60] Reddy AR 2003 The state of Rayalaseema. New Delhi, India: Mittal Publications.

[61] JRC. 2016 GHSL—Global Human Settlement Layer—European Commission. See http://ghsl.jrc.ec.europa.eu/.

[62] Taubenböck H, Roth A, Esch T, Felbier A, Müller A 2012 The vision of mapping the global urban footprint using the TerraSAR-X and TanDEM-X mission. In Urban and regional data management, pp. 243–251. London, UK: Taylor and Francis Group.

