# Supplementary material for: Exploring the high-resolution mapping of gender-disaggregated development indicators
Source: J R Soc Interface. 2017 Apr 5;14(129):20160825. doi: 10.1098/rsif.2016.0825 (PMC5414904; doi:10.1098/rsif.2016.0825)
Supplement: Supplementary Information [file rsif20160825supp1.pdf]

Submitted to the J. R. Soc. Interface

Exploring the high resolution mapping of gender disaggregated development indicators

Bosco C[1,2,3], Alegana V[1,2], Bird T[1,2], Pezzulo C[1,2], Bengtsson L[1,2,5], Sorichetta A[1,2], Steele J[1,2], Hornby, G[1], Ruktanonchai C[1,2], Ruktanonchai N[1,2], Wetter E [1,2,4], and Tatem AJ[1,2]

[1]WorldPop, Department of Geography and Environment, University of Southampton, Southampton, UK
[2]Flowminder Foundation, Stockholm, Sweden
[3]Department of civil and building engineering, Loughborough University, Loughborough, UK
[4]Stockholm School of Economics, Stockholm, Sweden
[5]Karolinska Institute, Dept. of Public Health Sciences, Stockholm, Sweden

Supplementary information (SI)

Table of Contents

1. Datasets
    1.1 Georeferenced DHS indicators
    1.2 Geospatial Covariates Layers
2. Materials and Methods
    2.1 Models specification
    2.2 Selection of geospatial covariate layers
    2.3 Semantic Array programming
3. Results
4. References

1 Datasets

The Demographic and Health Surveys (DHS) is a program of national household surveys implemented across a large number of LMICs. The DHS Program collects and analyses data on population demographic and health characteristics through more than 300 surveys in over 90 countries. The gender-disaggregated data we investigated in this report come from DHS datasets.

1.1 Georeferenced DHS indicators

In most DHS household surveys, a two (or three) stage cluster sampling procedure is adopted. Sampling clusters are usually the primary sampling units (PSU), which are pre-existing geographic areas known as census enumeration areas (EAs). A stratified sample of EAs is usually selected with probability proportional to size (PPS) (ICF International, 2012). The boundaries of the EAs are defined by the country's census bureau, as is the urban and rural status of each cluster. An EA can be a city block or apartment building in urban areas, while in rural areas it is typically a village or group of villages. At a second stage, within each identified EA, a fixed (or variable) number of households is selected and all eligible women and men in the selected households are interviewed (ICF International, 2012). The population and size of sampled clusters vary between and within countries. Typically, clusters contain 100-300 households, of which 20-30 households are randomly selected for survey participation (ICF International, 2012). The estimated center of each cluster is recorded as a latitude/longitude coordinate, obtained from a GPS receiver or derived from public online maps or gazetteers. The actual physical size or boundaries of the survey cluster are not made available. In recent years it has become more common for countries to use census EA boundary files to calculate the center of EAs (ICF International, 2012).

The georeferenced datasets can be linked to individual and household records in DHS household surveys through unique cluster identifiers. A displacement of up to 5 km in rural areas and up to 2 km in urban areas is introduced at the processing stage to anonymize cluster locations. Furthermore, around 1% of the rural clusters are displaced up to 10 km (Macro International Inc., 1996; Burgert et al. 2013; ICF International, 2012). The displacement affects the geolocation of the data, so this had to be taken into account when building the modelling dataset of indicators derived from the DHS survey. For linear modelling approaches, we used the mean value of each variable in a buffer of two kilometres from the cluster location for urban areas and five kilometres for rural areas, following published recommendations (Perez-Heydrich, 2013). For the non-linear modelling architectures (e.g. Artificial Neural Networks (ANN), the values came from a Monte Carlo analysis using the same buffers.

The publicly available DHS survey data and related cluster GPS coordinates for Kenya (2008-09) (KNBS, 2010), Tanzania (2010) (NBS, 2011), Nigeria (2013) (NPC, 2014), Bangladesh (2011) (NIPOR, 2013) and Haiti (2012) (Cayemittes et al., 2013) were obtained. The sections below describe the cluster level variables we mapped. We produced gender-disaggregated maps of nutrition, literacy and family planning indicators. These indicators are clearly defined by the DHS program and were constructed following the instructions contained in individual country final reports, and information from Rutstein and Rojas (2003).

1.2 Geospatial covariate layers

To predict each indicator at locations where survey data were not available, we exploited the relationship with covariate layers taking into account spatial autocorrelation (e.g. Alegana et al., 2015; Sedda et al., 2015). An important aspect of geostatistical modelling is the exploitation of geospatial covariates that are correlated with the outcome of interest, and can partially explain variation in that response, allowing for more accurate predictions across the map. A suite of covariates was chosen from existing publicly available libraries, based on factors that have previously been shown to correlate with development indicators in different settings (Alegana et al 2015, Gething et al 2015).

A set of several physical (topography, climate, land cover, etc.) and some social (population, ethnicity) covariate grids, completely covering the selected countries, were selected and tested as possible explanatory covariates across the analysed DHS indicators. With this being a short pilot project, because of the limited time and the long time necessary for assembling a proper set of covariates, differences among countries exists in the number and types of covariate grids in each of the datasets. These differences are mainly related to data availability and to the time necessary to build specific covariates when not already available. Due to the different spatial resolution, projection system, format and extent of the datasets, algorithms were developed and applied for converting all the layers in a common 1x1 km gridded datasets suitable to be used in map production. Information on features, sources, and naming conventions for each of the covariates are available in the following tables (1-4) and sub-sections.

Table 1 - Details of geospatial covariates assembled for use in mapping literacy and malnutrition in Bangladesh

Dataset name	Continuous or categorical	Data source	Year
Accessibility	Continuous	European Commission Joint Research Centre	2000
Population	Continuous	WorldPop	2010
Aridity Index	Continuous	CGIAR-CSI	1950-2000
Potential Evapotranspiration	Continuous	CGIAR-CSI	1950-2000
Cattle	Continuous	http://livestock.geo-wiki.org/	2006
Chickens	Continuous	http://livestock.geo-wiki.org/	2006
Ducks	Continuous	http://livestock.geo-wiki.org/	2006
Ruminant production System	Categorical	http://livestock.geo-wiki.org/	2006
Goats	Continuous	http://livestock.geo-wiki.org/	2006
Pigs	Continuous	http://livestock.geo-wiki.org/	2006
Sheeps	Continuous	http://livestock.geo-wiki.org/	2006
Nightlight	Continuous	NOAA VIIRS	2014
Elevation	Continuous	CGIAR-CSI (SRTM)	2003
MODIS EVI	Continuous	MODIS MOD13A1 [Enhanced vegetation index]	2010-2014
Distance to roads	Continuous	Input data from OSM (http://extract.bbbike.org/)	2014
Distance to waterways	Continuous	Input data from OSM (http://extract.bbbike.org/)	2014
Percentage of Urban Areas	Continuous	CIESIN - Global Rural Urban Mapping Project	2000
Protected Areas	Categorical	WDPA (http://www.protectedplanet.net/)	2012
Landcover	Categorical	ESA GlobCover Project	2009
Births	Continuous	WorldPop	2012
Pregnancy	Continuous	WorldPop	2012
Ethnicity	Categorical	ETH Zurich (http://www.icr.ethz.ch/data/geoepr)	2014
Precipitation	Continuous	WorldClim (http://www.worldclim.org)	1950-2000
Temperature	Continuous	WorldClim (http://www.worldclim.org)	1950-2000
Latitude	Continuous	Input data from worldpop	2015
Longitude	Continuous	Input data from Worldpop	2015

Table 2 - Details of geospatial covariates assembled for mapping literacy and stunting in Kenya

Dataset name	Continuous or categorical	Data source	Year
Accessibility	Continuous	European Commission Joint Research Centre	2000
Aridity index	Continuous	CGIAR-CSI	1950-2000
MID Infrared Index	Continuous	MODIS	2014
Nightlight	Continuous	NOAA VIIRS	2012
Settlement distance	Continuous	MODIS 500m Global Urban Extent	2000/2001
Temperature (annual mean)	Continuous	WorldClim/BioClim (BIO1)	1950-2000
MODIS EVI	Continuous	MODIS MOD13A1 [Enhanced vegetation index]	2014
Population	Continuous	WorldPop Project	2010
Distance to roads	Continuous	Input data from OSM (http://extract.bbbike.org/)	2015
Elevation	Continuous	GEOTOPO (SRTM like at ~1km)	2000
Potential evapotranspiration	Continuous	CGIAR-CSI	1950-2000
Percentage of urban areas	Continuous	MODIS 500m Global Urban Extent	2000/2001
Precipitation (annual mean)	Continuous	WorldClim (http://www.worldclim.org)	1950-2000

Table 3 - Details of geospatial covariates assembled for mapping literacy, malnutrition and the use of modern contraception methods in Nigeria

Dataset name	Continuous or categorical	Data source	Year
Accessibility	Continuous	European Commission Joint Research Centre	2000
Aridity index	Continuous	CGIAR-CSI	1950-2000
MID Infrared Index	Continuous	MODIS	2001-2005
Nightlight	Continuous	NOAA VIIRS	2012
Settlement distance	Continuous	Input data from "WorldPop Project"	2010
Temperature	Continuous	MODIS	2001-2005
MODIS EVI	Continuous	MODIS MOD13A1 [Enhanced vegetation index]	2001-2005
Population	Continuous	WorldPop Project	2010
Distance to roads	Continuous	Input data from OSM (http://extract.bbbike.org/)	2014
Elevation	Continuous	USGS	2001

Potential evapotranspiration	Continuous	CGIAR-CSI	1950-2000
Percentage of urban areas	Continuous	Input data from ”WorldPop Project”	2010
Cattle	Continuous	FAO/ERGO	2005
Distance to conflicts	Continuous	Input data from ACLED	2010-2013
Goats	Continuous	FAO/ERGO	2005
Gross cell production	Continuous	Yale G-Econ Research Project	2006-2008
Protected areas	Categorical	WDPA (http://protectedplanet.net/)	2012
Pigs	Continuous	FAO/ERGO	2005
Poultry	Continuous	FAO/ERGO	2005
Distance to rivers	Continuous	Input data from VMAP0	Multiple years
Sheeps	Continuous	FAO/ERGO	2005
Small ruminant	Continuous	FAO/ERGO	2005
Rainfed crop suitability	Categorical	FAO FGGD	2005
Landcover	Categorical	NASA/USGS	2011

Table 4 - Details of geospatial covariates assembled for mapping literacy and use of modern contraception methods in Tanzania

Dataset name	Continuous or categorical	Data source	Year
Accessibility	Continuous	European Commission Joint Research Centre	2000
Aridity index	Continuous	CGIAR-CSI	1950-2000
MID Infrared Index	Continuous	MODIS	2014
Nightlight	Continuous	NOAA VIIRS	2012
Settlement distance	Continuous	MODIS 500m Global Urban Extent	2000/2001
Temperature (mean annual)	Continuous	WorldClim/BioClim (BIO1)	1950-2000
MODIS EVI	Continuous	MODIS MOD13A1 [Enhanced vegetation index]	2014
Population	Continuous	WorldPop Project	2010
Distance to roads	Continuous	Input data from OSM (http://extract.bbbike.org/)	2015
Elevation	Continuous	GEOTOPO (SRTM like at ~1km)	2000
Potential evapotranspiration	Continuous	CGIAR-CSI	1950-2000
Percentage of urban areas	Continuous	MODIS 500m Global Urban Extent	2000/2001
Precipitation (mean annual)	Continuous	WorldClim (http://www.worldclim.org)	1950-2000

In order to test the value of inclusion of these spatial datasets as covariates in the statistical models, the following steps were taken:

1) The study area was rasterized at a resolution of 30 arc-seconds (i.e., 0.00833333 degree corresponding to ~ 1km at the equator)

2) Vector datasets were rasterized at a resolution of 30 arc-seconds

3) If needed, raster datasets were resampled to 30 arc-seconds using the most appropriate interpolation technique, depending on the category, type, and original spatial resolution of each given dataset

4) Finally, all datasets were co-registered (i.e., spatially aligned so that all pixels representing the same area and location in different datasets are exactly coincidental) and matched to the rasterized study area (by clipping them to the study area and/or filling their "NoData" pixels located within the study area)

1.2.1 Accessibility

The EU Joint Research Center maintains a gridded surface that estimates accessibility, measured in likely travel times (via all transport methods), to cities with more than 50,000 inhabitants. In practice, this provides a useful composite measure of the extent to which regions are rural or urban, as well as the degree of their connectedness to the national system of transportation. Areas near major roads, for example, would be relatively well connected, even if they were some distance from major cities. More details of this geospatial layer can be found at http://forobs.jrc.ec.europa.eu/products/gam.

1.2.2 Elevation

A digital elevation model (DEM) derived from the NASA Shuttle Radar Topography Mission (SRTM) Near-global Digital Elevation Models (DEMs) was obtained for all the countries: http://webmap.ornl.gov/wcsdown/wcsdown.jsp?dg_id=10008_1.

1.2.3 Nightlights

Visible Infrared Imaging Radiometer Suite (VIIRS) composite nightlight imagery for 2012 and 2014 were obtained (http://ngdc.noaa.gov/eog/). These surfaces allow differentiation of regions based on the density of population and the degree of electrification of dwellings, commercial and industrial premises, and infrastructure.

1.2.4 Population density

As part of the WorldPop project (www.worldpop.org.uk), gridded data on population density were created from settlement, land use and other geospatial data derived from satellite imagery, and used to disaggregated areal census population counts to 100x100m grid squares.

1.2.5 MODIS Enhanced Vegetation Index (EVI)

EVI composites are produced globally at 500-metre resolution on 16-day intervals from data collected by the MODIS sensor on the Terra satellite. MODIS EVI products are computed from atmospherically corrected bi-directional surface reflectances that have been masked for water, clouds, heavy aerosols, and cloud shadows. These data are used for global monitoring of vegetation condition (Thome et al). For this study, 16-day composites were downloaded for the most recent 5 years (2010 – 2014) and averaged at the pixel level to create a long-term average of vegetation condition.

1.2.6 MODIS Mid-infrared (MIR) reflectance

MIR reflectance is the surface reflectance in the middle-infrared part of the electromagnetic spectrum. MIR reflectance (2105 – 2155 nm) is collected globally at 500-metre resolution every 16 days by the MODIS sensor on the Terra satellite. These data are used for characterising biophysical properties of the land surface. Vegetation spectral signatures are characterised by low reflectance in the visible and MIR (controlled by pigment and water absorption features). For this study, 16-day composites were downloaded for the years 2001 – 2005 and averaged at the pixel level to create a long-term average of MIR reflectance.

1.2.7 Livestock densities

Data from Gridded Livestock of the World v2.0 were obtained via the Livestock Geo-Wiki (http://livestock.geo-wiki.org) (Robinson, 2014). Distribution maps of livestock densities at 1 km resolution were downloaded for cattle, pigs, ducks, goats, sheep, and chickens.

1.2.8 Aridity and Potential Evapotranspiration (PET)

The CGIAR Consortium maintains high-resolution global raster climate data related to evapo-transpiration processes and rainfall deficit for potential vegetative growth. These are based on data from the WorldClim project and ultimately from weather station data that has been interpolated by using covariates such as altitude. These grids, extracted for four of the countries, allowed for differentiation of areas with adequate rainfall and moisture regimes to sustain agriculture as opposed to areas where drier and more arid conditions prevail (http://csi.cgiar.org/Aridity/).

1.2.9 Urban/Rural and distance to settlements

For this study the urban extents grid for the year 2000, produced by Columbia University Centre for International Earth Science Information Network (CIESIN) was obtained. The grid distinguishes urban and rural areas based on combining population counts, settlement points, and nighttime lights (CIESIN, 2011). We also exploited a settlement location vector dataset produced within the framework of the WorldPop Project (refer to Tatem et al., 2004; 2005 for a description of the methodology used to produce the dataset) that was used to calculate some of the "Distance to settlements" variables. In Tanzania and Kenya we used the MODIS 500m Global Urban Extent to calculate the distance to human settlement. It is based on Moderate Resolution Imaging Spectroradiometer (MODIS) 500-m satellite data and is freely available through https://nelson.wisc.edu/sage/data-and-models/schneider.php.

In Haiti we used the Global Human Settlement Layer (GHSL) with a resolution of 38m, to calculate the distance per pixel to the boundary of the nearest human settlement, for the year 2015. For pixels located within urban areas the 'distance to' value is presented as a negative value, i.e. the distance to the outer urban boundary, thereby providing additional variability. GHSL data is produced within the framework of the European Commission JRC Global Human Settlement Analysis for Disaster Risk Reduction project, implemented by the EC JRC Global Security and Crisis Management Unit. These data are provided to WorldPOP as pre-public-release for scientific research purposes only.

1.2.10 Protected areas

Protected areas were obtained from the World Database on Protected Areas (WDPA), available through (IUCN & UNEP-WCMC, 2012).

1.2.11 Births

Birth data estimating the number of live births per 100-metres were downloaded from the WorldPop Project (http://www.worldpop.org.uk/). The estimates are adjusted to match UN national estimates on numbers of live births (Tatem et al., 2014). For this study we aggregated the 2012 estimates of number of live births per pixel to 1-kilometre.

1.2.12 Pregnancies

Pregnancy data estimating the number of pregnancies per 100-metres were downloaded from the WorldPop Project (http://www.worldpop.org.uk/). The estimates were adjusted to match national estimates on numbers of pregnancies made by the Guttmacher Institute[6]. For this study we aggregated the 2012 estimates of number of pregnancies per pixel to 1-kilometre.

1.2.13 Ethnicity

Geocoded ethnic groups were obtained via the GeoEPR 2014 dataset available through ETH Zurich. These data identify politically relevant ethnic groups in every country from 1946 to 2013, including annual data on more than 800 groups (Vogt et al., 2015).

1.2.14 Bioclimatic variables

We used global climate layers produced by WorldClim for annual trends from 1950 – 2000 for temperature and precipitation. BIO1 is the annual mean temperature, calculated by the mean of all the weekly mean temperatures. Each weekly mean temperature is the mean of that week's maximum and minimum temperature. BIO12 is the annual precipitation, calculated as the sum of all the monthly precipitation estimates (Hijmans et al., 2005).

1.2.15 Distance to conflicts

Locations of conflicts between 2010 and 2013 in Nigeria were extracted from the ACLED (Armed Conflict Location and Event Data Project) database Version 4 (1997 – 2013)

(http://www.acleddata.com/data/acled-versions-1-5-data-1997-2014/) and used to calculate the "Distance to conflict" variables.

1.2.16 Gross cell production

Gross cell production was derived from a geophysical-based data set on economic activity (representing the regional equivalent of gross domestic product) measured at a 1-degree longitude by 1-degree latitude resolution at a global scale (http://gecon.yale.edu/data-and-documentation-g-econ-project). Each pixel (cell) contains a gross product value calculated by accounting for economic data merged with other important demographic and geophysical data including climate, physical attributes, location indicators, population, and luminosity (refer to Nordhaus et al. 2006 for a detailed description of derivation of G-Econ data).

1.2.17 Rainfed crop suitability

Rainfed crop suitability was derived from a global raster dataset with a resolution of 5 arc-minutes (http://www.fao.org:80/geonetwork/srv/en/resources.get?id=14172&fname=Map6_61.zip &access=private). Each pixel contains the suitability index for rainfed crops, calculated using maximising crop and technology mix (2005 version), while pixels representing urban areas, closed forests, and irrigated areas contain negative values.

1.2.18 Land cover

The IGBP MODIS Land Cover Type product (MCD12Q1), with a native resolution of about 500m, is derived from input of Terra- and Aqua-MODIS data and includes 11 natural vegetation classes, 3 developed and mosaicked land classes, and three non-vegetated land classes (refer to Friedl et al., 2010 for a detailed description of how the MCD12Q1 dataset was produced).

1.2.19 Distance to roads

For all countries, the "Distance to roads" covariate was calculated using the most recent Open Street Map road network dataset (http://extract.bbbike.org/) available at the time of analysis.

1.2.20 Distance to waterways

The "Distance to waterways" covariate was calculate either using the most recent Open Street Map river network dataset (http://extract.bbbike.org/) available at the time of analysis or the corresponding VMAP0 dataset (http://geoengine.nga.mil/geospatial/SW_TOOLS/NIMAMUSE/webinter/rast_roam.html) depending on which one was more detailed for each country (refer to tables 1-4 above).

1.2.21 Distance to schools

The “Distance to schools” covariate was calculated using the most recent Open Street Map point location dataset (http://extract.bbbike.org/) available at the time of analysis.

1.2.22 Distance to health facility

The “Distance to health facility” covariate was derived from Open Street Map point location data. In this instance “Health Facility” locations relate to Cholera Treatment Centres and Cholera Treatment Units recorded up until Dec 2011, extracted from http://haitidata.org/.

2 Materials and Methods

2.1 Models specification

Bayesian model specification

The Gaussian Function (GF) in INLA is represented as a Gaussian Markov Random Function (GMRF). Computations in INLA are carried out using the GMRF by approximating a set of spatial-temporal random function with weighted sum of basis functions. The advantage of computation using the GMRF as approximations to GF with Matérn covariance is due to the Markovian property of the former resulting in sparse matrices that are computationally efficient. The explicit link between the continuous domain GF is a solution to the stochastic partial differential equation (SPDE) expressed as:

$$\frac{\partial}{\partial t}\left(k^2-\Delta\right)^{\alpha/2}\left(\tau x(s)\right)=w(s)$$

Where $\left(k^2-\Delta\right)^{\alpha/2}$ is a differential operator, k is a scaling parameter, Δ is the Laplacian, α controls the smoothness of realization, τ controls the variance and $x(s)$ is the spatial-temporal domain for s(s1,……,sn) locations. $w(s)$ is the Gaussian white noise. A stationary model was implemented. The link between Matérn smoothness ν and variance σ^2 is given by $\alpha=\nu+d/2$ where d is spatial dimension and marginal variance $\sigma^2=1/\left(\Gamma(\nu)\Gamma(\alpha)(4\pi)^{d/2}k^{2\nu}\tau^2\right)$. The above SPDE is solved via finite element method by triangulating the domain of interest. The SPDE is projected to a basis representation,

$$x(s)=\sum_k \psi_k(s)x_k$$

Several advantages arise from such construction. First, the aforementioned resulting sparse covariance matrix is computationally efficient since GMRF is only discretely indexed at locations x1,……xk. Secondly the covariance function is flexible within the general family of covariances. The stationary Matérn covariance function for spatial lag distance $h=\|s_1-s_2\|$, s1 and s2 locations is expressed as:

$$C(s_1s_2)=\frac{\sigma^2}{2^{\nu-1}\Gamma(\nu)}(kh)^{\nu}k_{\nu}(kh)$$

Where k_ν is the modified Bessel function of second order and h is the Euclidean distance while σ^2 is the marginal variance.

The SPDE generates precision matrix of form $Q=Q_t\otimes Q_s$ where Q_s is the precision in the spatial domain and Q_t in temporal domain with $Q_s=\tau^2\left(k^4C+2kG_1+G_2\right)$ where C, G1 and

G2 are sparse matrices while Q_t precision was based on a temporal AR(1) process similar to Cameletti et al. (2012).

A linear model was implemented using gaussian, zero-inflated binomial or beta likelihood for the proportion of literacy, stunting and use of modern contraception methods.

$$z(s) = x(s)\beta + \zeta(s) + \varepsilon(s)$$

Where $z(s)$ are realizations of the process linked to a structured predictor in an additive way, $x(s)$ denotes set of covariates with β coefficients and $\varepsilon(s)$ is the measurement error while $\zeta(s)$ represent first order autoregressive dynamics with spatially correlated innovations.

Gaussian likelihood were adopted in the majority of the case; beta likelihood were applied for modelling male literacy in Kenya and the use of modern contraception methods in Tanzania and zero-inflated binomial for modelling the use of modern contraception methods in Nigeria.

Artificial Neural Networks specification

An artificial neuron is a computational model inspired by natural neurons. Natural neurons receive signals through synapses located on the dendrites or membrane of the neuron. When the signals received are strong enough (surpass a certain threshold), the neuron is activated and emits a signal though the axon. This signal might be sent to another synapse, and might activate other neurons.

The complexity of real neurons is highly abstracted when modelling artificial neurons. These basically consist of inputs (like synapses), which are multiplied by weights (strength of the respective signals), and then computed by a mathematical function which determines the activation of the neuron. Another function computes the output of the artificial neuron .
An ANN is implemented by a system of interconnected nodes. Information propagates through nodes transforming the inputs in intermediate derived signals up to generate the final outputs. The internal nodes are called neurons and define the ANN hidden layers. Each node is a processing element propagating weighted inputs received from other nodes (Pradhan, 2009) (Fig. 5.4).

The higher the weight of an artificial neuron, the stronger the input which is multiplied by it will be. Weights can also be negative, thus we can say that the signal is inhibited by the negative weight. Depending on the weights, the computation of the neuron will be different. By adjusting the weights of an artificial neuron we can obtain the output we want for specific inputs.

Depending on the specific ANN architecture, the inputs of a given node may include or exclusively be constituted by intermediate derived signals. The learning process comes from adjusting the weights between neurons analysing the error between the predicted and target output. The output of a neural network, after the training, is a model that is capable of predicting a target value from an input dataset (Lee, 2007).

A useful class of ANN applications (Bosco et al., 2013; de Rigo et al., 2001; Secomandi, 2000) is based on the multilayer feedforward networks (multilayer perceptrons) due to their universal approximation properties (Kreinovich, 1991; Hornik et al., 1989). A feedforward neural network is an ANN having connections between the different units not forming a cycle or loop. In this architecture information moves in only one direction, from the input nodes, through the hidden layers (if any) to the output nodes. The main reason for the use of this type of ANN is the simplicity of its theory, ease of programming and good results (Fig. 5.5 shows the scheme of this kind of ANN).

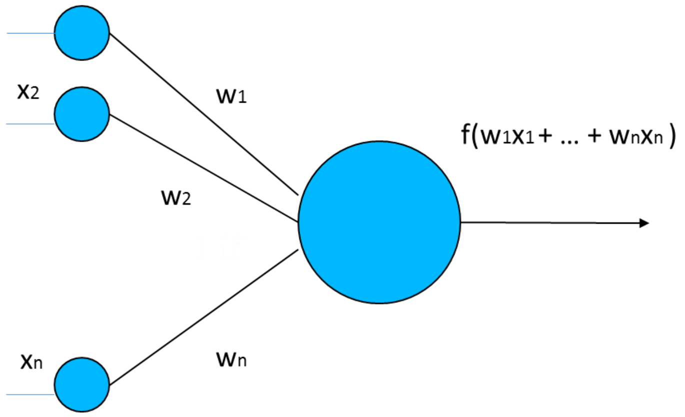

Fig. 5.4 – Artificial neuron model. Inputs to the network are represented with the symbol x_n, each of these inputs are multiplied by a connection weight w_n, summed and fed through the transfer function f() to generate a result and the output. This configuration is actually called a Perceptron. The perceptron (an invention of Rosenblatt (1962)), was one of the earliest neural network models.

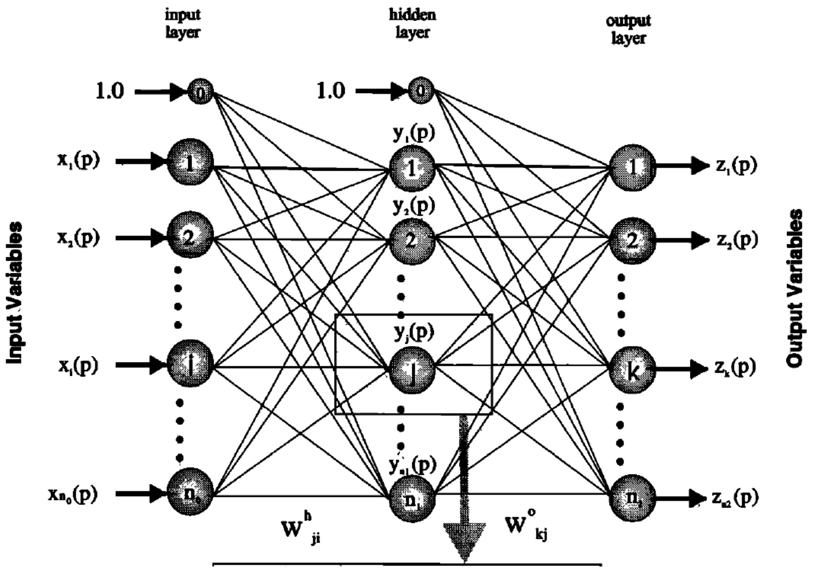

Figure 5.5: A generic single layer feedforward neural network (Ghassemieh and Nasseri, 2012).

Each of the processing neurons calculates the weighted sum of all interconnected signals from the previous layer plus a bias term and then produce an output through the activation function. The effective incoming signal $s_j$to node j is:

$$s_j = \sum_{i=0}^{n_0} w_{ij} x_i$$

The activation function associating individual nodes have typically a sigmoid shape (Fig. 5.6). The sigmoid function most often used for ANNs is the logistic function:

$$y_j = f(s_j) = 1/[1 + \exp(-s_j)]$$

in which s_j can vary in the range ±∞ but y is bounded between 0 and 1.

Other transfer functions can also be applied. The adjustment of the ANN function to experimental data (training of the network) is based on a non-linear regression procedure (Fraser, 2000). Random weights are assigned to each neuron, the output of the network is evaluated and the error between the output of the network and the training dataset is calculated. If the error is large, the weights are adjusted and the process goes back to evaluate the network's output. This cycle is repeated until the error is small or a stop criterion is satisfied.

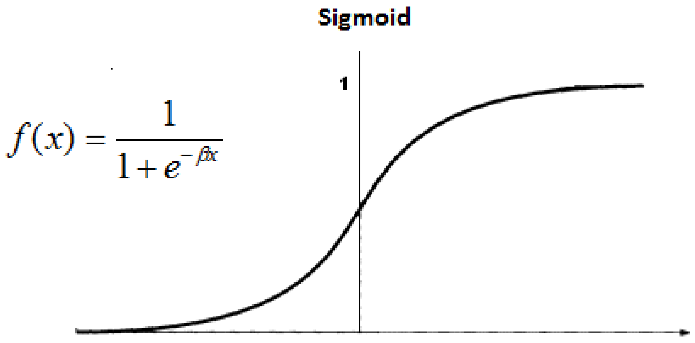

Fig. 5.6 Example of a sigmoid function

During the training of a neural network, the prediction error is evaluated at each iteration. The use of an ANN with too many neurons allows an excess of degrees of freedom. This can cause overfitting of the data.

A test dataset can be separated and used to check how good the prediction capacity of the ANN is, on the basis of the sum of squared prediction errors. For obtaining the optimal degree of training it is possible to explore the ANN performance in order to minimize the sum of the training plus validation (or cross-validation) errors (Figure 5.7), whilst ensuring that the training process is not stopped at the first point of minimum. Training should be allowed to proceed further to check whether or not it is a point of local minimum, since a local minimum can be found.

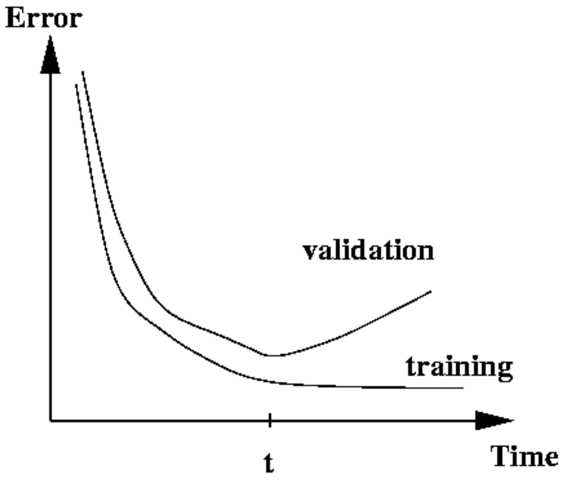

Fig. 5.7 – Profiles for training and validation errors

Within the present study we explored the use of feed-forward neural networks through the package AMORE (A MORE flexible neural network) (Castejón Limas et al., 2014) in GNU R and through the package Neural Network (Schmid, 2009) available in GNU Octave (Eaton, 2008). The functions in these packages allow to develop the most common type of neural network model (the feed-forward multi-layer perceptron). The functions have enough flexibility to allow the user to develop the best or most optimal models by varying parameters during the training process.

In the package AMORE, the user can select the number of layers and the number of neurons in each layer, while controlling several parameters. These include the learning rate at which every neuron is trained, the momentum for every neuron, the error criterion (least mean squares, least mean logarithm squares or TAO), the activation function of the hidden and the output layer (Purelin, Tansig, Sigmoid, or Hardlim), and the training method (Adaptive gradient descent or BATCH gradient descent, with or without momentum).

The same principle applies for the package Neural Network where the user can select the number of layers and neurons in each layer and the activation function of the hidden and the output layers (Purelin, Tansig, logsig) to apply with the training algorithm (Levenberg-Marquardt).

With these parameters selected, the algorithm trains the network with the manually or randomly selected samples before testing it with the rest of the samples.

Feed-forward neural networks provide a flexible way also to generalize linear regression functions. They are non-linear regression models but with many parameters, such that they are extremely flexible-flexible enough to approximate any smooth function. (Venables and Ripley, 2002).

2.2 Selection of geospatial covariate layers

For obtaining a more appropriate combination of covariates to produce high-resolution prediction maps for each of the modelled indicators, a sensitivity analysis using a jackknife approach was carried out (Fig.1). The jackknife analysis consists of dropping one observation at a time from one set of data, and calculating the estimate each time. It was developed by Maurice Quenouille, (1949, 1956) and John Tukey (1958) expanded on the technique and proposed the name "jackknife".

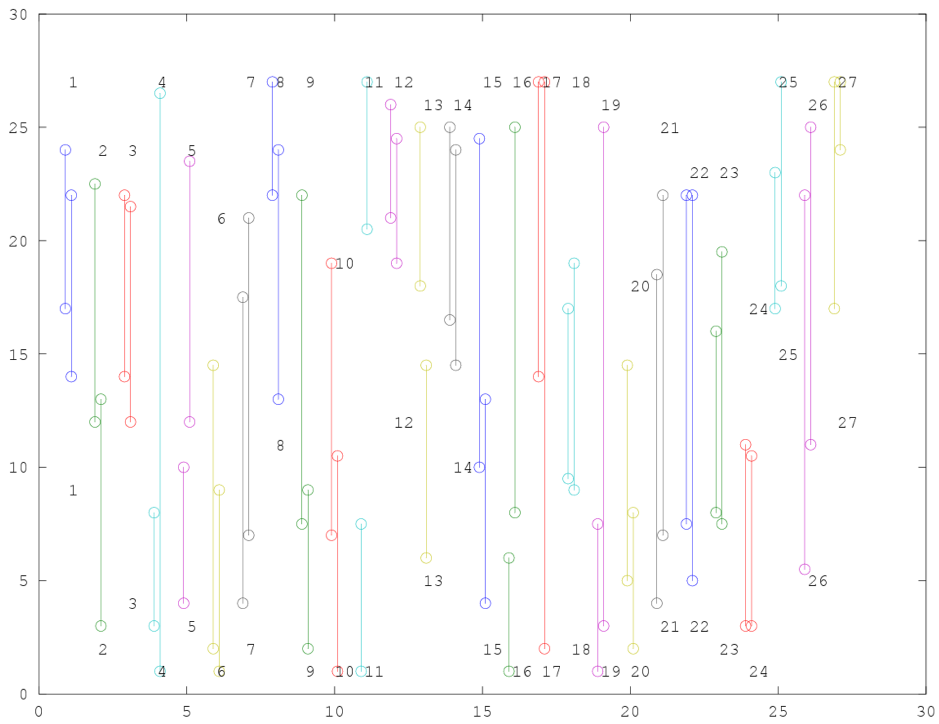

Fig. 1 – Results of the jackknife analysis implemented in MATLAB language in GNU Octave for selecting the final subset of covariates of an artificial neural network. The x-axis shows the subset of covariates where the ith covariate was removed. The y-axis is the score obtained by comparing the MSE of all the subset of covariates in each of the 400 runs of the jackknife loop. The two bars represent the results with one and two neurons, respectively. In this example, the 12th covariate is a good candidate to be removed both in the ANN with one and two neurons.

Within the modelling architectures, categorical covariates with more than two levels were recoded into a number of separate dichotomous variables in order for the results to be interpretable. All covariates were also normalized to make all variables have a mean of zero and unit variance.

2.3 Semantic Array programming

Managing heterogeneous arrays of data and data transformation models in a systematic and structured way is a challenging task. The multiplicity of model families, covariates and modelled quantities in this work required the support of a common, flexible and scalable modelling architecture. The applied modelling architecture is based on the Semantic Array Programming (SemAP) paradigm (de Rigo, 2012; 2015). Array programming (AP) emerged as a way to reduce the gap between mathematical notation and algorithm implementations by promoting arrays (vectors, matrices, tensors) as atomic quantities with compact manipulating operators (Iverson, 1980). Atomicity here implies that even a large array of data is managed

as a single logical piece of information. For example, a regional-scale gridded layer may be managed by AP languages as if it were a single variable instead of a large matrix of elements.A disciplined use of AP (Iverson, 1980) may allow nontrivial data-processing to be expressed with very concise expressions (Taylor, 2003) and a potentially simpler control flow.
However, this capability for abstraction and simplification of AP may be limited by the very same generality of AP data structures-multi-dimensional arrays where the value of some elements may be infinite or not-a-number (IEEE 754 standard) or even complex-valued (de Rigo, 2015). The Semantic Array Programming paradigm has been introduced for supporting a disciplined semantics-aware implementation of AP concepts and methods, with additional systematic semantic checks for the semantic correctness of the chain of modelling blocks (de Rigo, 2012).

This is why our computational modelling methodology follow the SemAP paradigm by combining concise implementation of the model with its conceptual subdivision in semantically enhanced abstract modules.

Two additional design concepts (de RIgo, 2012) define the SemAP paradigm as supported by the Mastrave library:

1. The modularisation of sub-models (and more generally of any autonomous task which displays a nontrivial extent of complexity) with a systematic effort toward their most concise generalization and reusability in other contexts.
2. The use of semantic constraints (based on the mathematics of arrays and defined following the physical/conceptual meaning of the modelled quantities) with array oriented –thus concise– invariants applied to the information entered in and returned by each module. Even this design pattern is applied systematically *within* each SemAP module instead of relying on external assumptions (which might prove more fragile in case of, for example, subsequent refactoring of a certain chain of data transformations where key external checks are inadvertently removed).

Modularization also expects consistent code self-documentation and uniform predictable conventions for module interfaces (without directly interfering with the preferred module's implementation). Semantic constraints contribute to enforce, within each module, autonomous distributed consistency checks instead of assuming top-down correctness of input information (e.g. instead of relying on object-oriented “monolithically-designed-to-be-safe” data).

3 Results

This section presents the results for the gender-disaggregated indicator mapping addressed in this project. We organize the presentation of results by indicator, at gender disaggregated level, in the following order: literacy, stunting in children, use of modern contraception methods. For each indicator, the results of a first exploratory analysis are presented with gender disaggregated histograms showing the basic statistical distribution of the indicator at cluster level and a scatter plot of the predicted versus observed data both in training and validation. We then present the results of the covariate selection exercise, detailing which covariates were selected as the optimum performing set for the given indicator for each country at gender disaggregated level and, for each indicator having an associated modelling explained variance higher than 0.3, we show maps of the survey clusters and the indicator value at each cluster, maps of the predicted proportion of modelled indicators and the level of uncertainty associated with these maps in each pixel, and finally the quantile-quantile (Q-Q) plot in training and validation. The maps reported in the following paragraphs are: male and female literacy rate in Nigeria and Kenya, female literacy rate in Tanzania, male and female stunting in Nigeria and the proportion of women using modern contraception methods in Nigeria and Tanzania.

3.1 Literacy

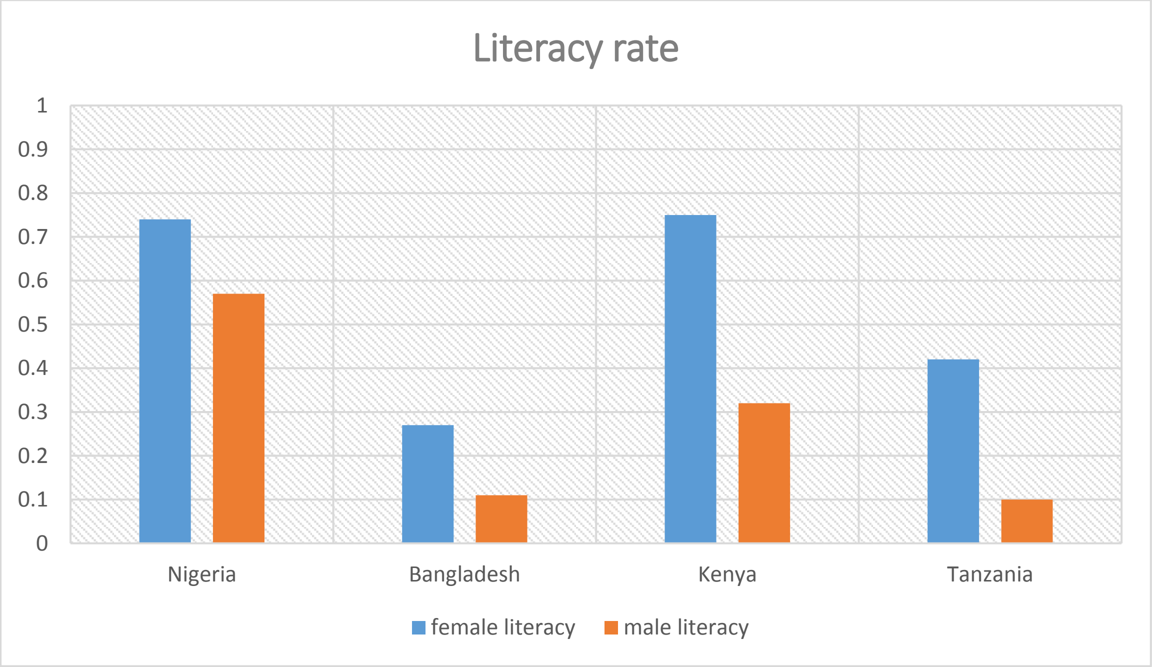

Fig 2. Explained variance of the gender disaggregated literacy maps in Nigeria, Bangladesh, Kenya and Tanzania.

3.1.1 Nigeria literacy

Figure 3a. Histograms (top row) related to the distribution of a 60% subset of DHS data (training) and scatter plot of the predicted (y-axis) and observed (x-axis) proportion of literacy for women and men age 15-49 in the training (middle row) and validation (bottom row) dataset.

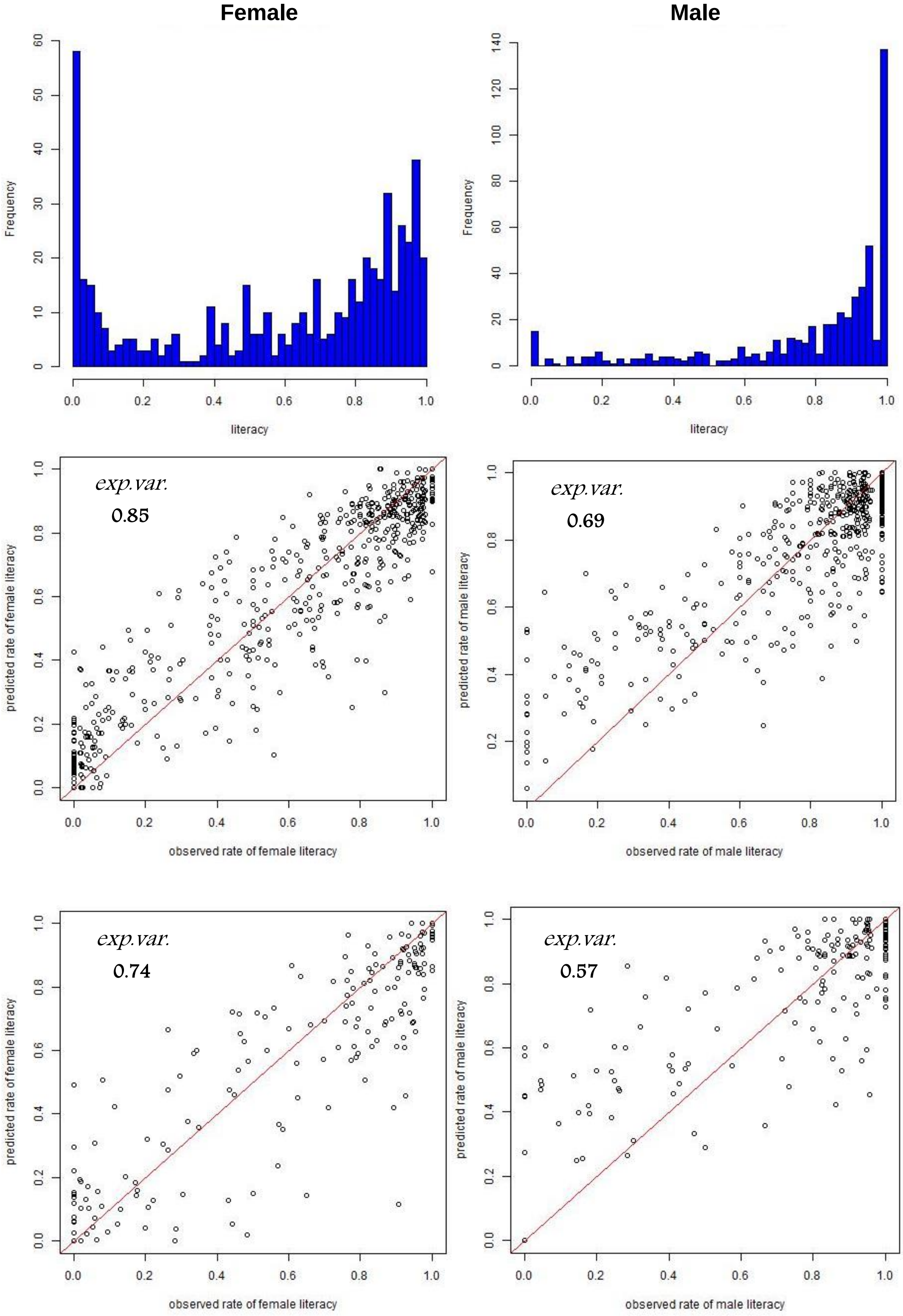

Figure 3b. Q-Q plot of the predicted (y-axis) and observed (x-axis) proportion of literacy for women and men age 15-49 in the training (top row) and validation (bottom row) dataset.

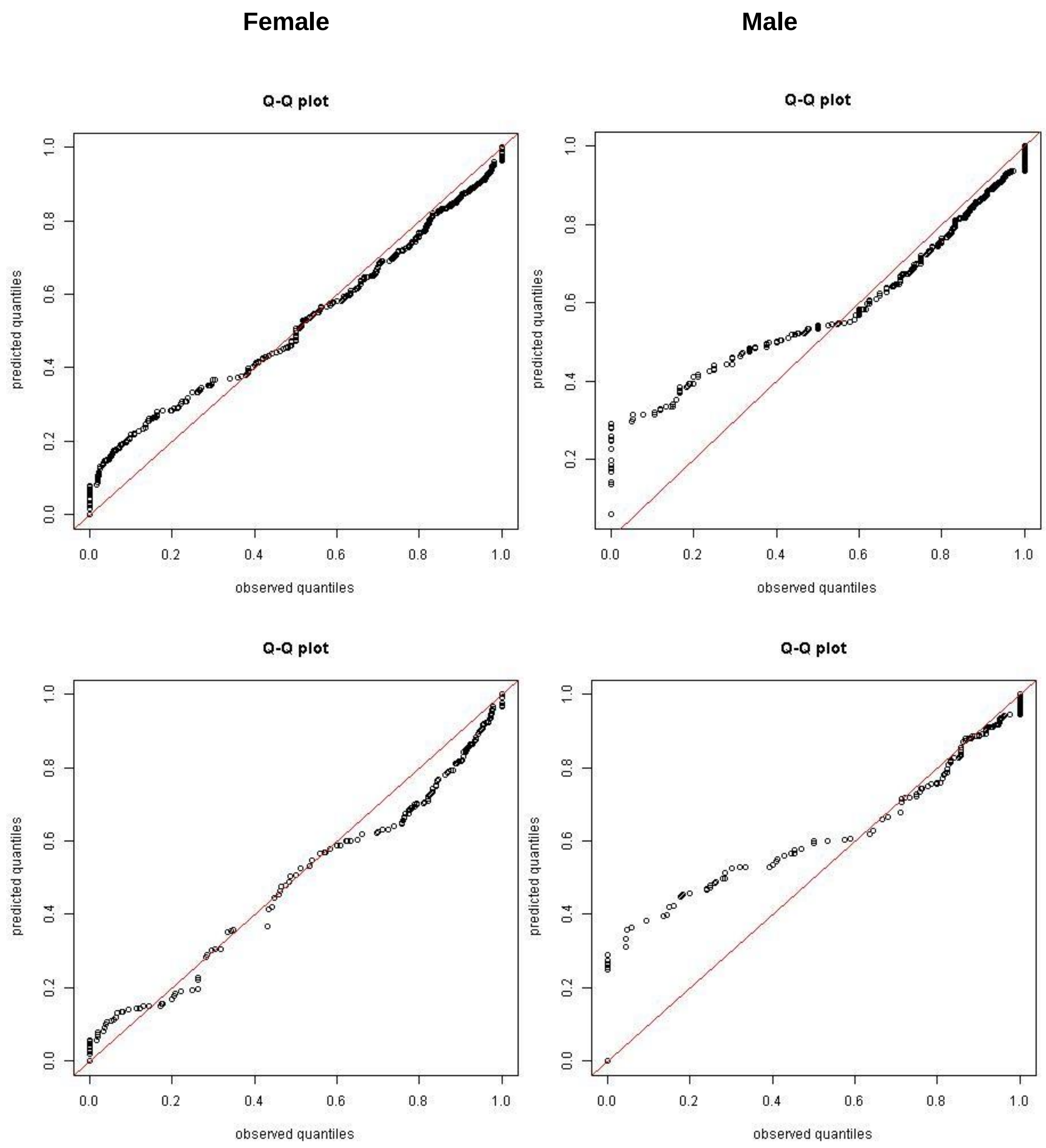

Figure 4. Map of the cluster-level survey data (top row), for the indicator on literacy of men and women aged 15-49. Map of the mean predicted proportion of literacy for women and men at 1 km^2 resolution (middle row) and related uncertainty maps (bottom row) showing their standard deviation.

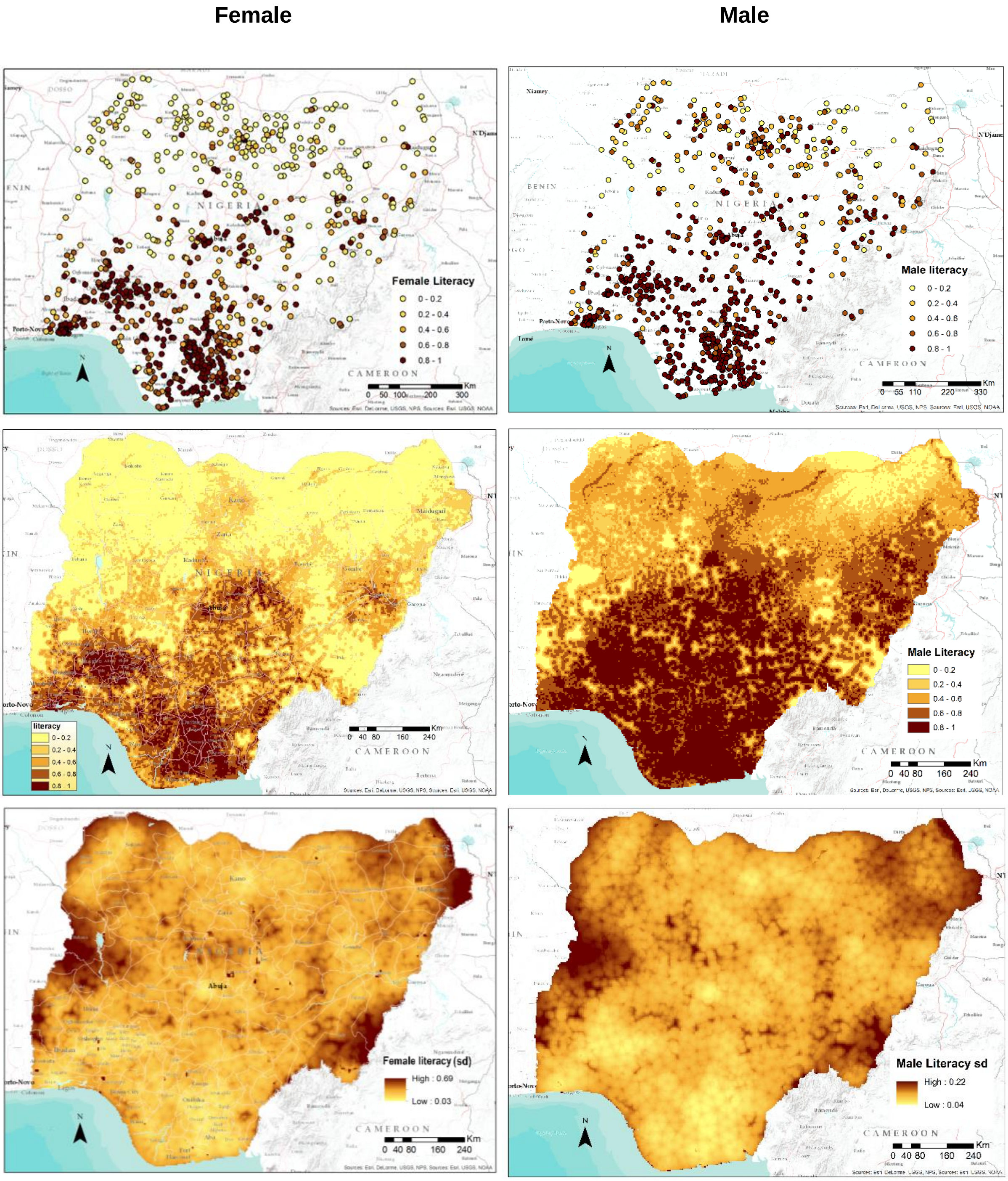

3.1.2 Kenya literacy

Figure 5a. Gender disaggregated histograms (top row) related to the distribution of a 70% subset of DHS data (training) and scatter plot of the predicted (y-axis) and observed (x-axis) proportion of literacy for men and women age 15-49 in the training (middle row) and validation (bottom row) dataset.

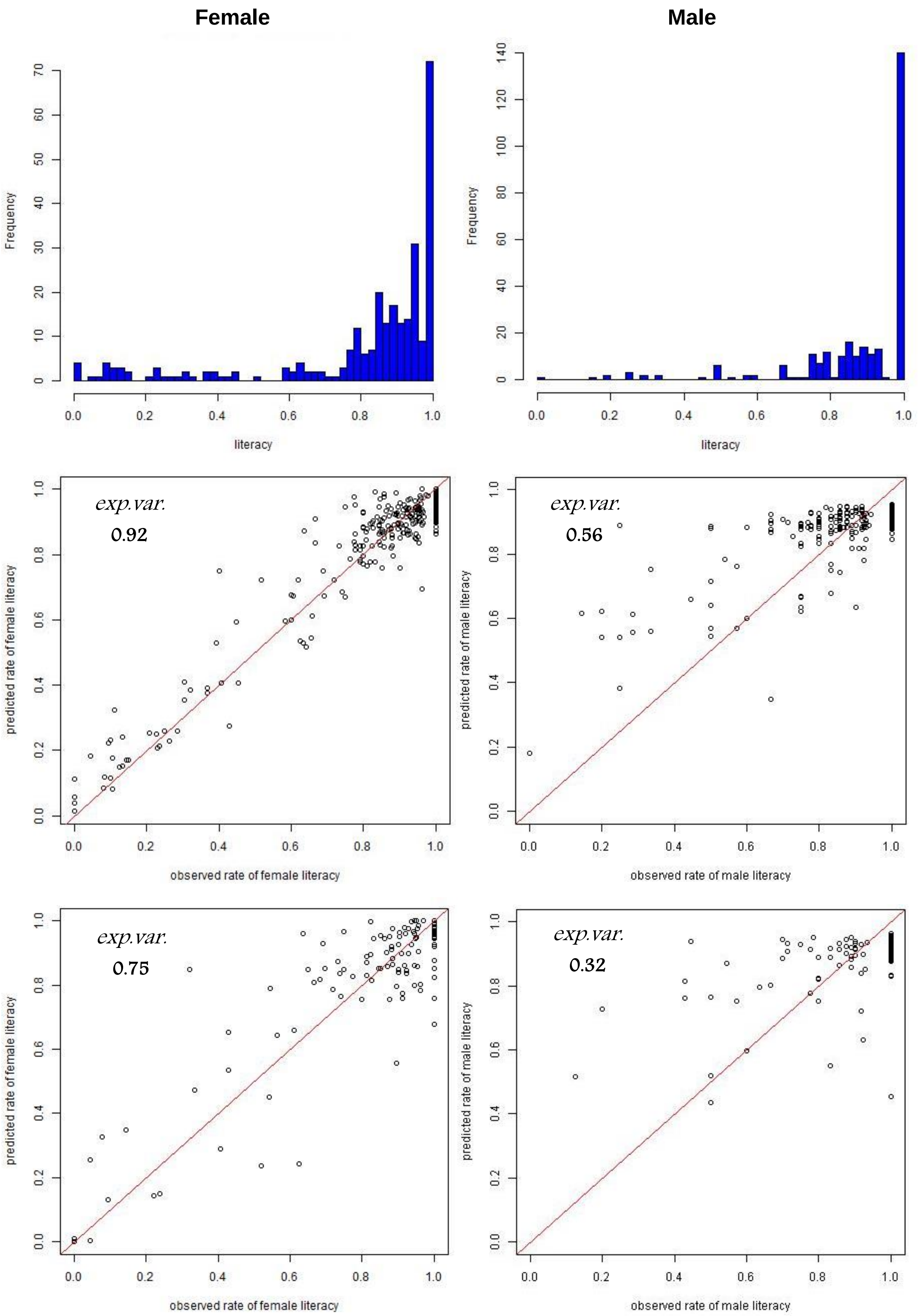

Figure 5b. Q-Q plot of the predicted (y-axis) and observed (x-axis) proportion of literacy for women and men age 15-49 in the training (top row) and validation (bottom row) dataset.

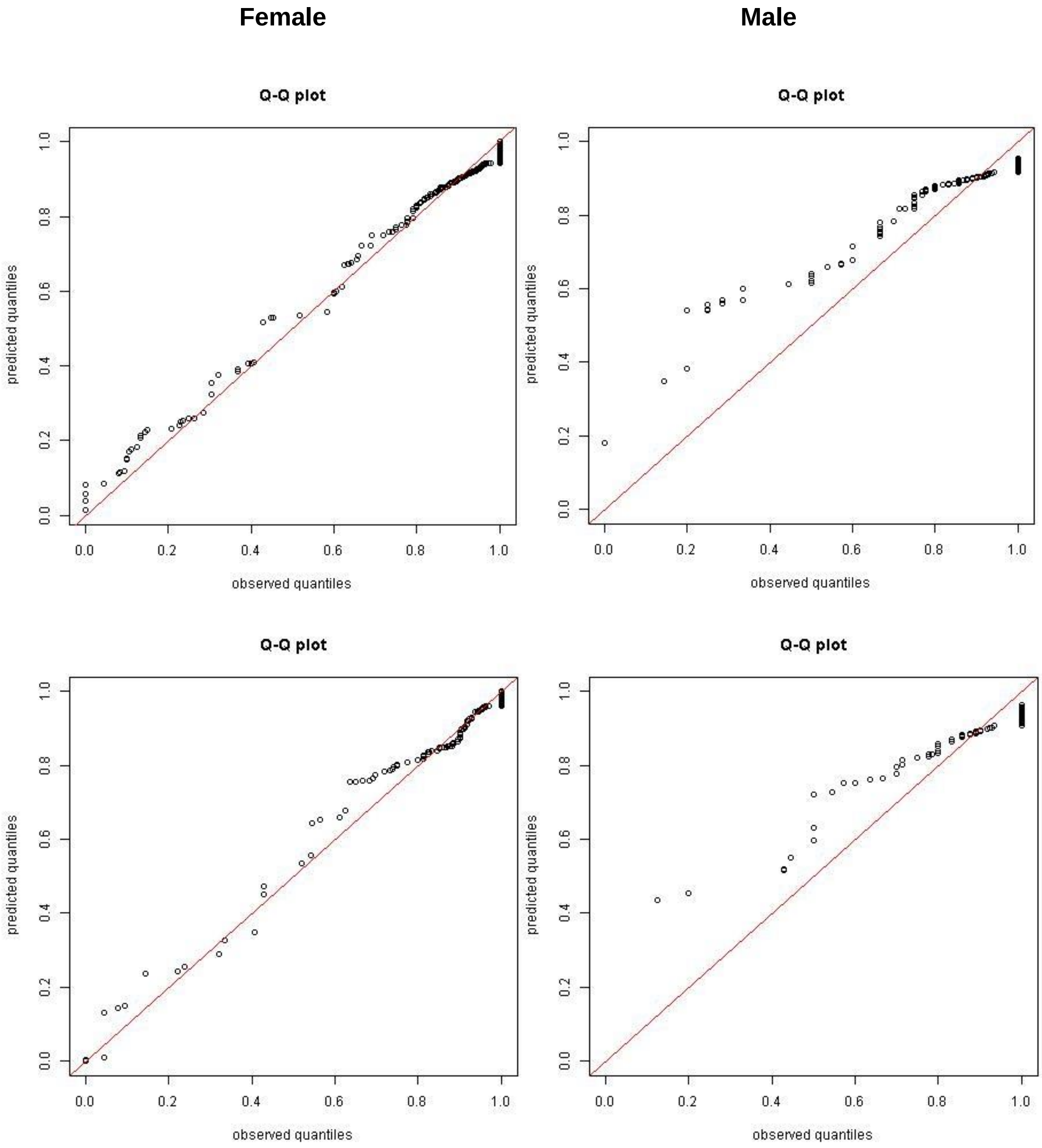

Figure 6. Map of the cluster-level survey data (top row), for the indicator of literacy of men and women aged 15-49. Map of the mean predicted proportion of literacy for women and men at 1 km^2 resolution (middle row) and related uncertainty maps (standard deviation) (bottom row).

Female **Male**

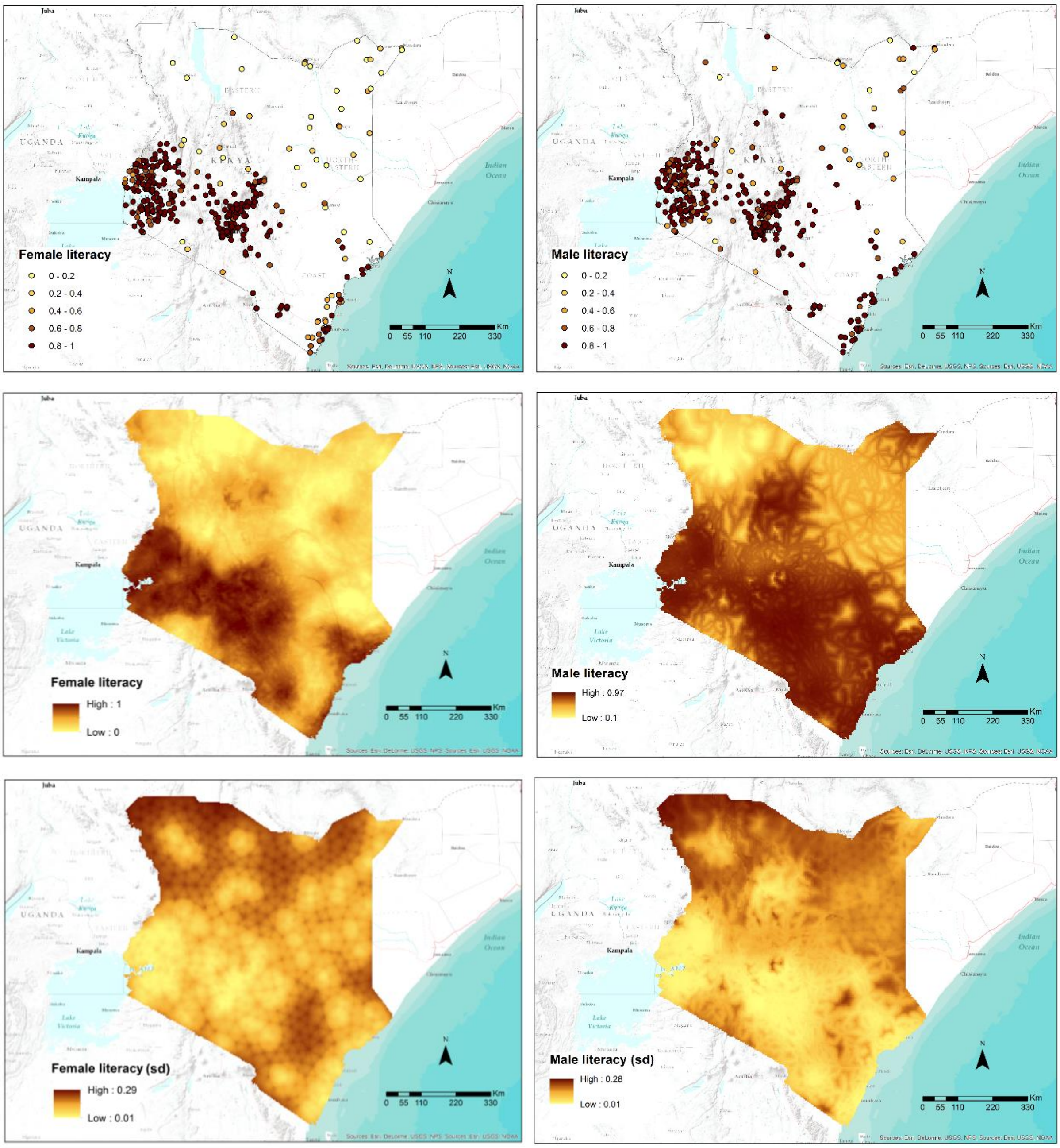

3.1.3 Tanzania literacy

Figure 7a. Gender disaggregated histograms (top row) related to the distribution of a 70% subset of the data (training) and scatter plot of the predicted (y-axis) and observed (x-axis) proportion of literacy for men and women age 15-49 in training (middle row) and validation (bottom row) dataset.

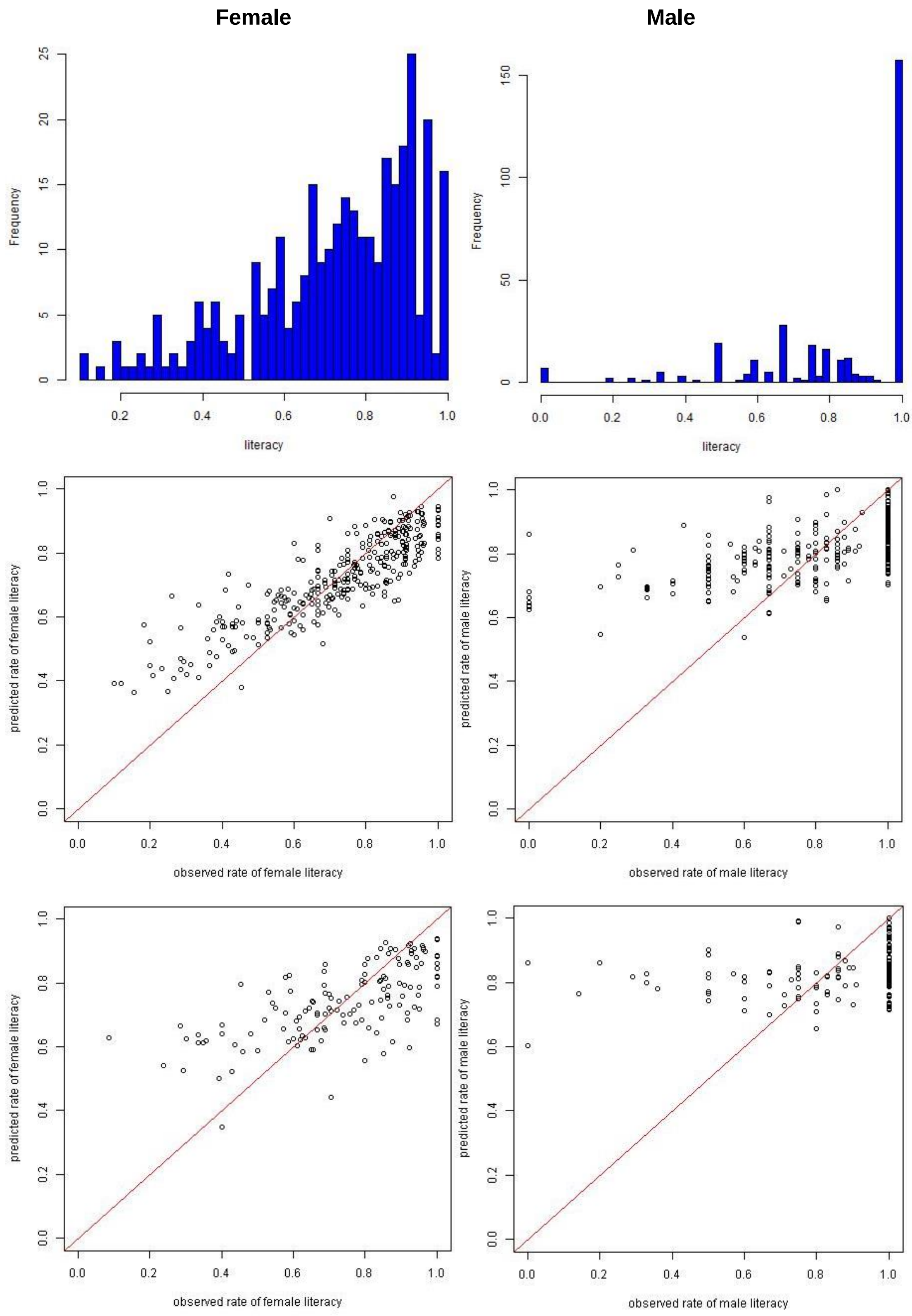

Figure 7b. Q-Q plot of the predicted (y-axis) and observed (x-axis) proportion of literacy for women and men age 15-49 in the training (top row) and validation (bottom row) dataset.

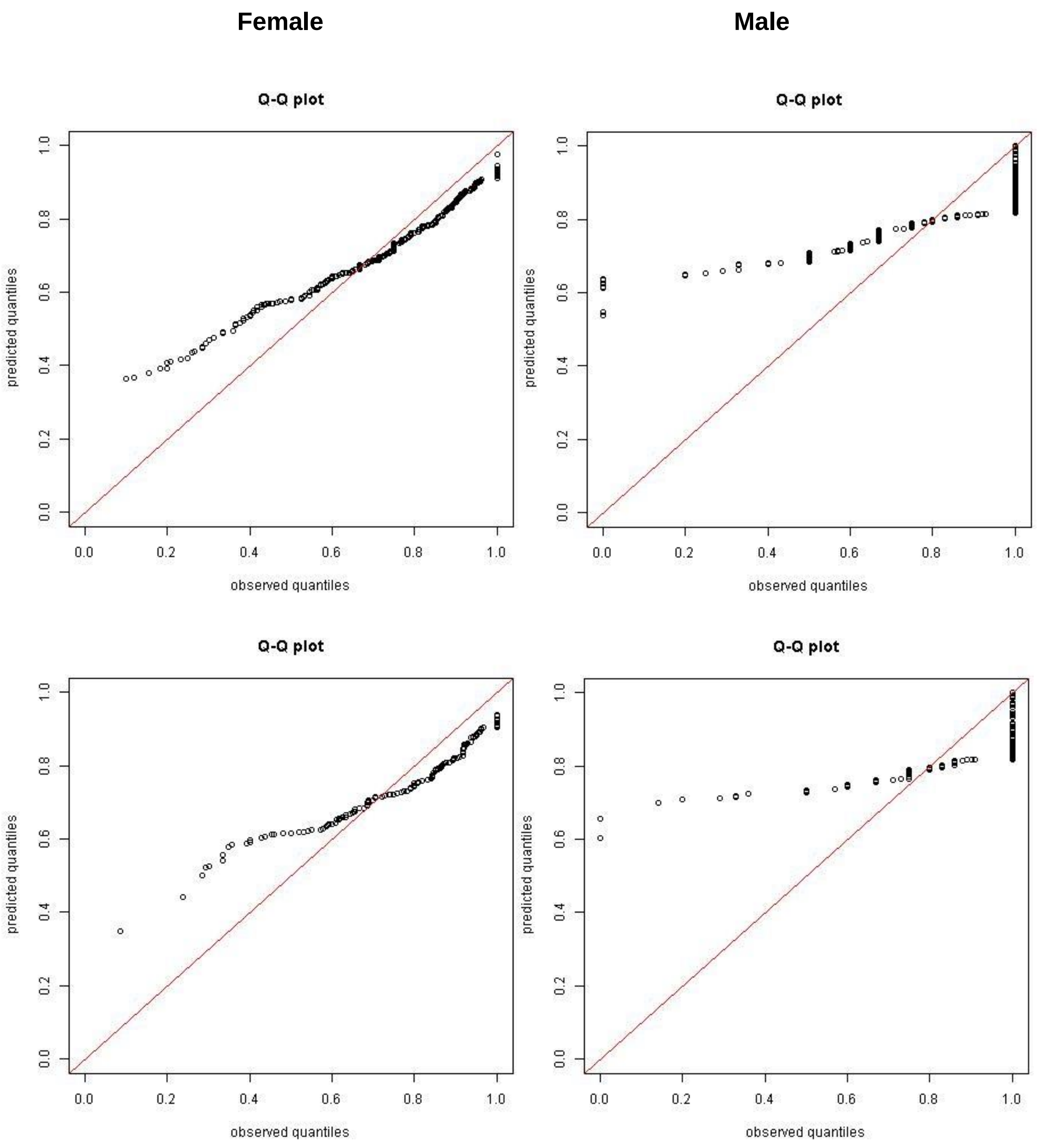

Figure 8. Map of the cluster-level survey data (top row) for the indicator of literacy of women aged 15-49. Map of the mean predicted proportion of literacy for women at 1 km^2 resolution (middle row) and related uncertainty map (standard deviation) (bottom row).

Female

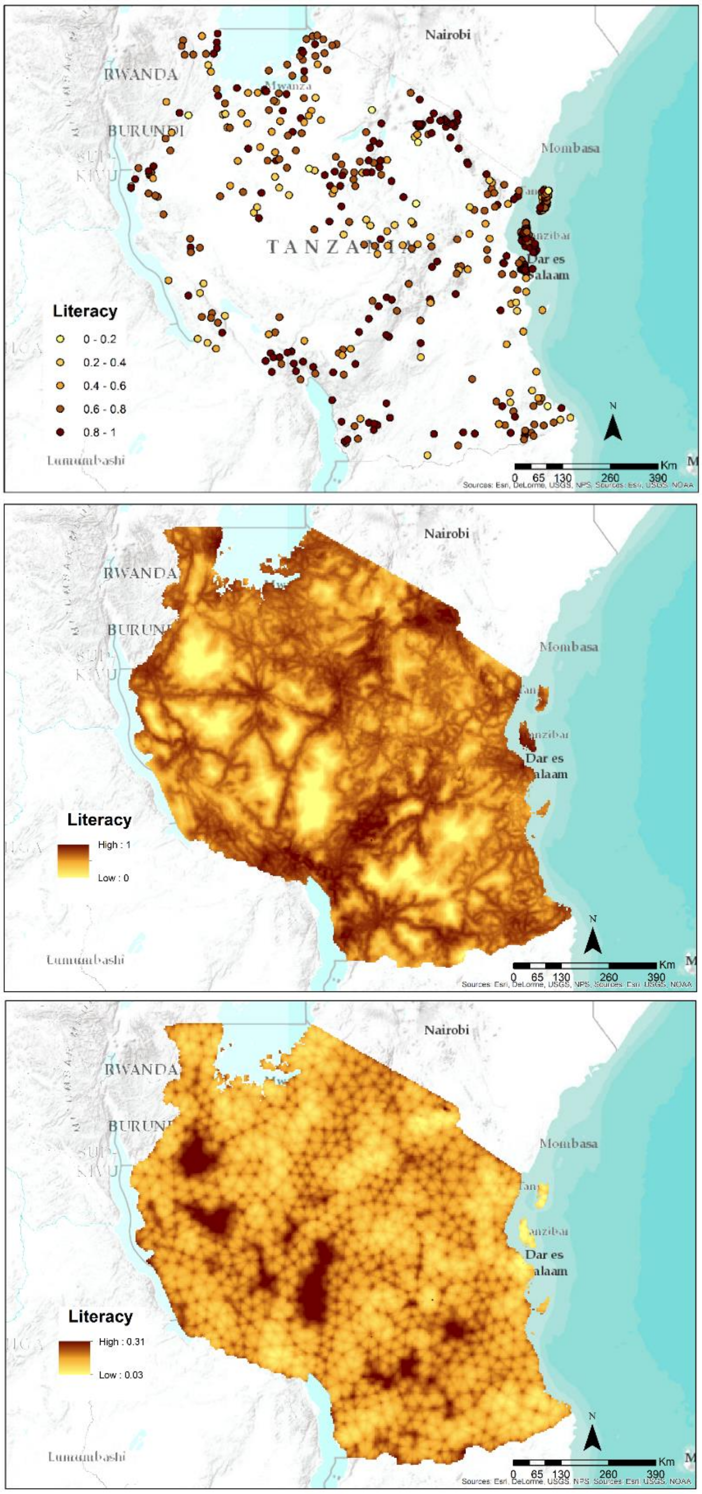

3.1.4 Bangladesh literacy

Figure 9. Gender disaggregated histograms (top row) related to the distribution of a 70% subset of the data (training) and scatter plot of the predicted (y-axis) and observed (x-axis) proportion of literacy for men and women age 15-49 in training (middle row) and validation (bottom row) dataset.

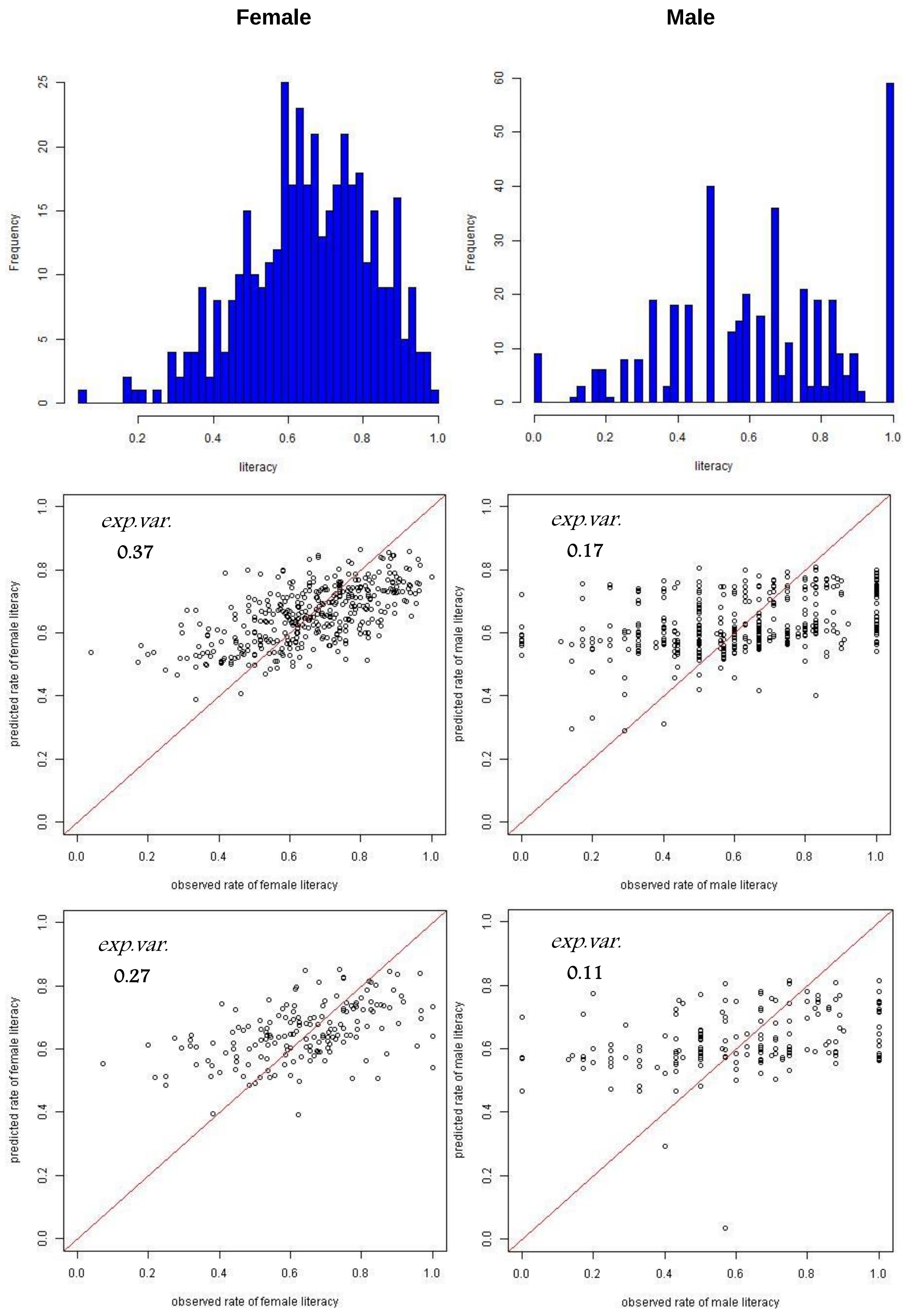

3.1.5 Literacy Covariate Selection

Table 5. Summary output from the covariate selection procedure for the indicator on male and female literacy in Nigeria, Kenya, Tanzania and Bangladesh. *Exp. Var.* is the proportion of variance explained by each of the models.

Nigeria	Female	Male
**N. of covariates**	7	7
**Exp. Var.**	0.74	0.57
**Selected Covariates**	Distance to conflicts<br>Elevation<br>MID Infrared Index<br>Settlement distance<br>Cattle<br>Pigs<br>Accessibility	Distance to conflicts<br>Elevation<br>MID Infrared Index<br>Settlement distance<br>Gross cell production<br>Population<br>Temperature

Kenya	Female	Male
**N. of covariates**	7	6
**Exp. Var.**	0.75	0.32
**Selected Covariates**	Potential Evapotranspiration<br>MID Infrared Index<br>Accessibility<br>Settlement distance<br>Nightlight<br>Elevation<br>Precipitation	Potential Evapotranspiration<br>MID Infrared Index<br>Accessibility<br>Settlement distance<br>Aridity index<br>Temperature

Tanzania	Female	Male
**N. of covariates**	8	8
**Exp. Var.**	0.42	0.10
**Selected Covariates**	Aridity index<br>Accessibility<br>Precipitation<br>Distance to roads<br>Nightlight<br>MID Infrared Index<br>Settlement distance<br>Temperature	Aridity index<br>Accessibility<br>Precipitation<br>Distance to roads<br>Nightlight<br>MODIS EVI<br>Latitude<br>Elevation

Bangladesh	Female	Male
**N. of covariates**	10	12
**Exp. Var.**	0.27	0.11
**Selected Covariates**	Percentage of Urban areas<br>Accessibility<br>Ducks<br>Distance to waterways<br>Potential Evapotranspiration<br>Goats<br>Population<br>Ethnicity<br>Latitude<br>Longitude	Percentage of Urban areas<br>Accessibility<br>Ducks<br>Distance to waterways<br>Distance to roads<br>Temperature<br>Cattle<br>Pigs<br>Sheeps<br>Births<br>Precipitation<br>Protected areas

3.2 Stunting

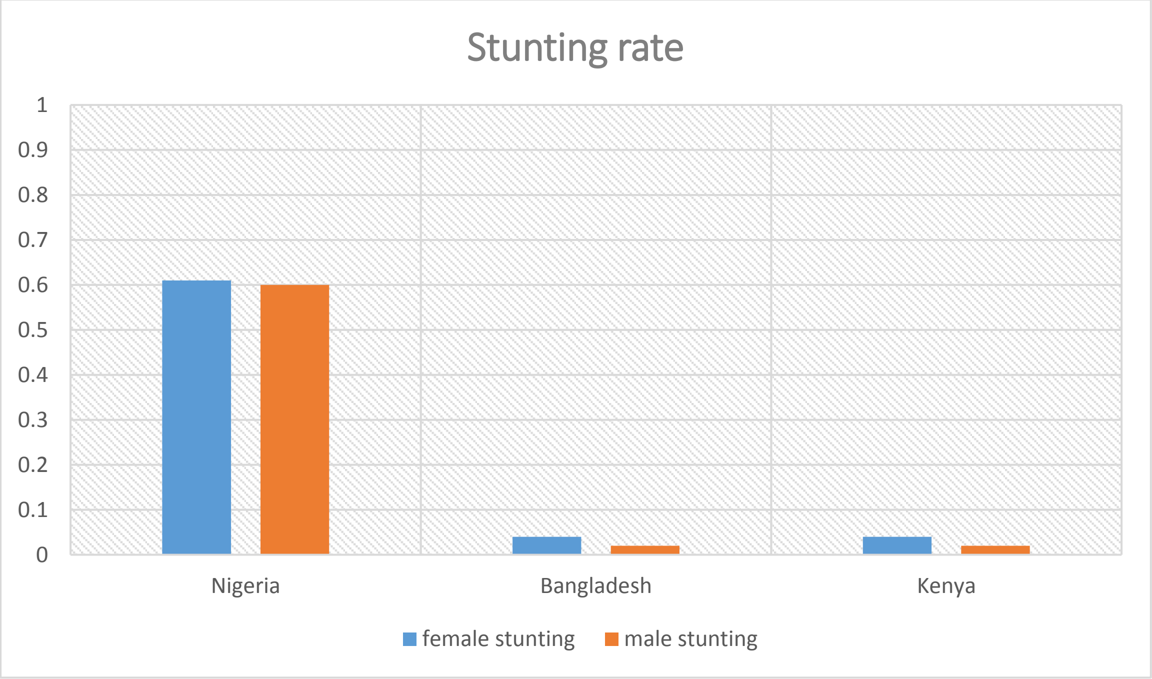

Fig 10. Explained variance of the gender disaggregated maps of stunting in children in Nigeria, Bangladesh and Kenya.

3.2.1 Nigeria stunting

Figure 11a. Histograms (top row) related to the distribution of a 60% subset of DHS data (training) and scatter plot of the predicted (y-axis) and observed (x-axis) proportion of stunting for children under the age of 5 in the training (middle row) and validation (bottom row) dataset.

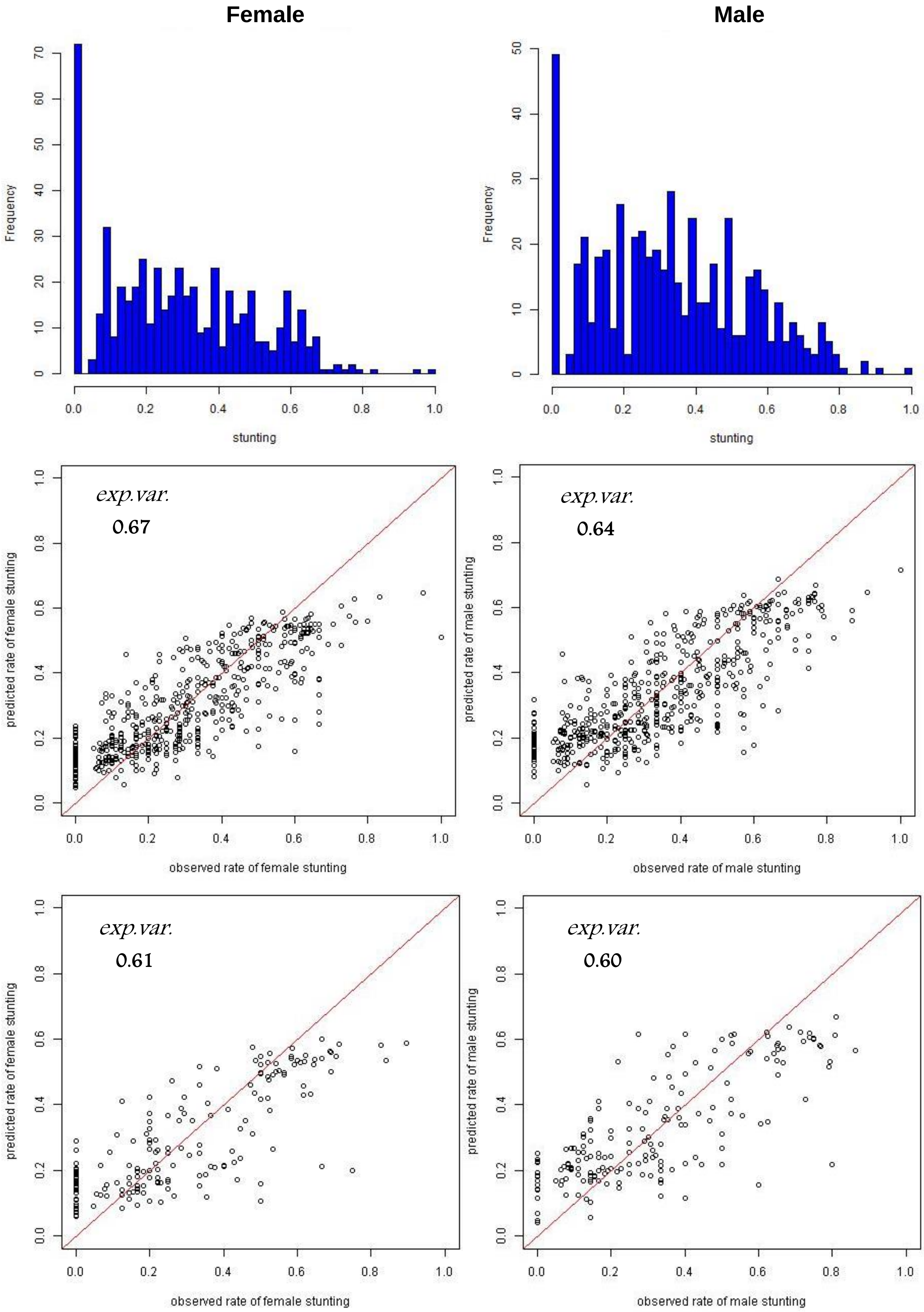

Figure 11b. Q-Q plot of the predicted (y-axis) and observed (x-axis) proportion of stunting for children under age of 5 in the training (top row) and validation (bottom row) dataset.

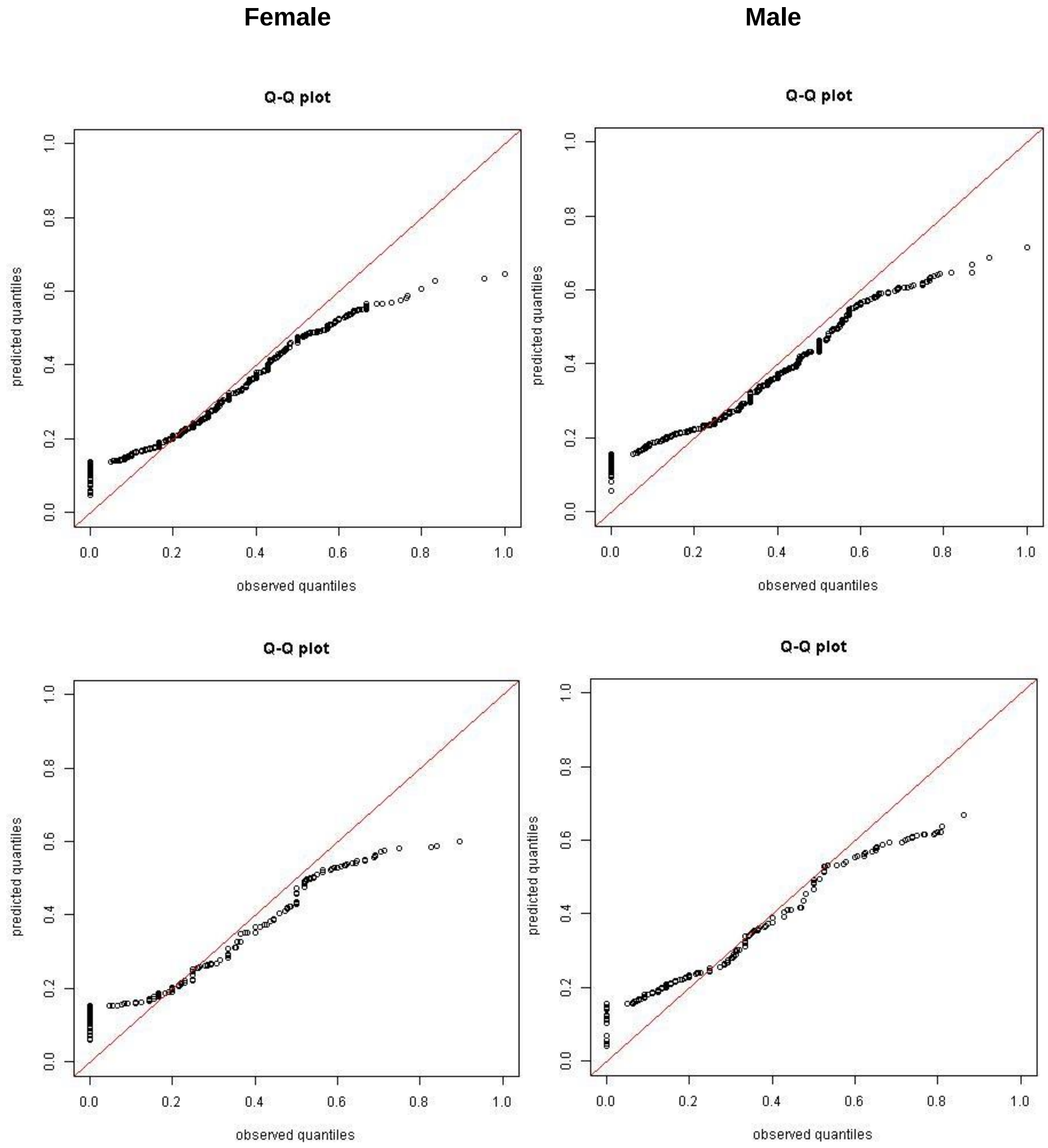

Figure 12. Map of the cluster-level survey data (top row), for the indicator on stunting of male and female children under the age of 5. Maps of the mean predicted proportion of stunting at 1 km^2 resolution (middle row) at gender disaggregated level and related uncertainty maps (bottom row) showing the associated interdecile range.

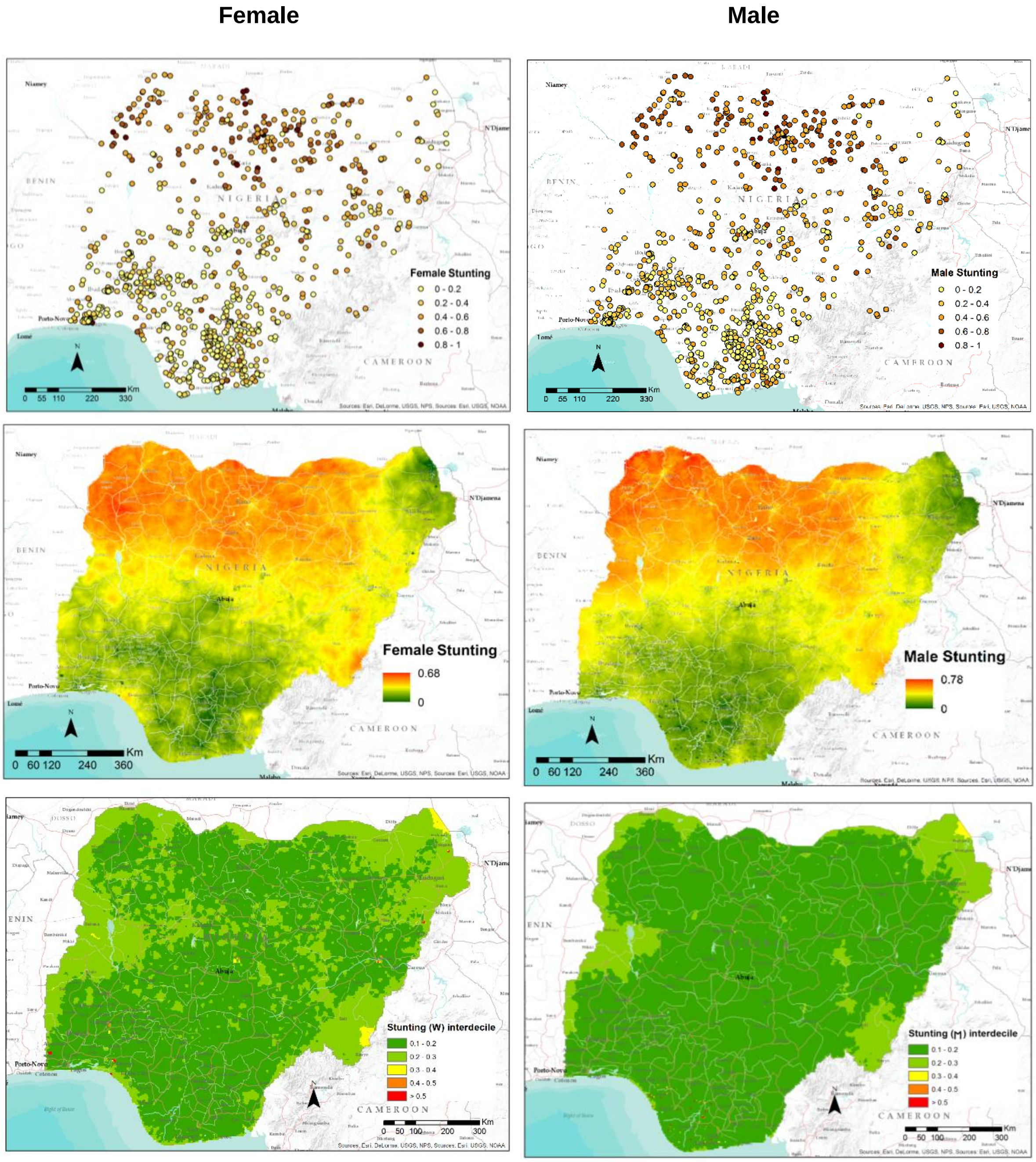

3.2.2 Kenya stunting

Figure 13. Gender disaggregated histograms (top row) related to the distribution of a 70% subset of DHS data (training) and scatter plot of the predicted (y-axis) and observed (x-axis) proportion of stunting for children under the age of 5, in the training (middle row) and validation (bottom row).

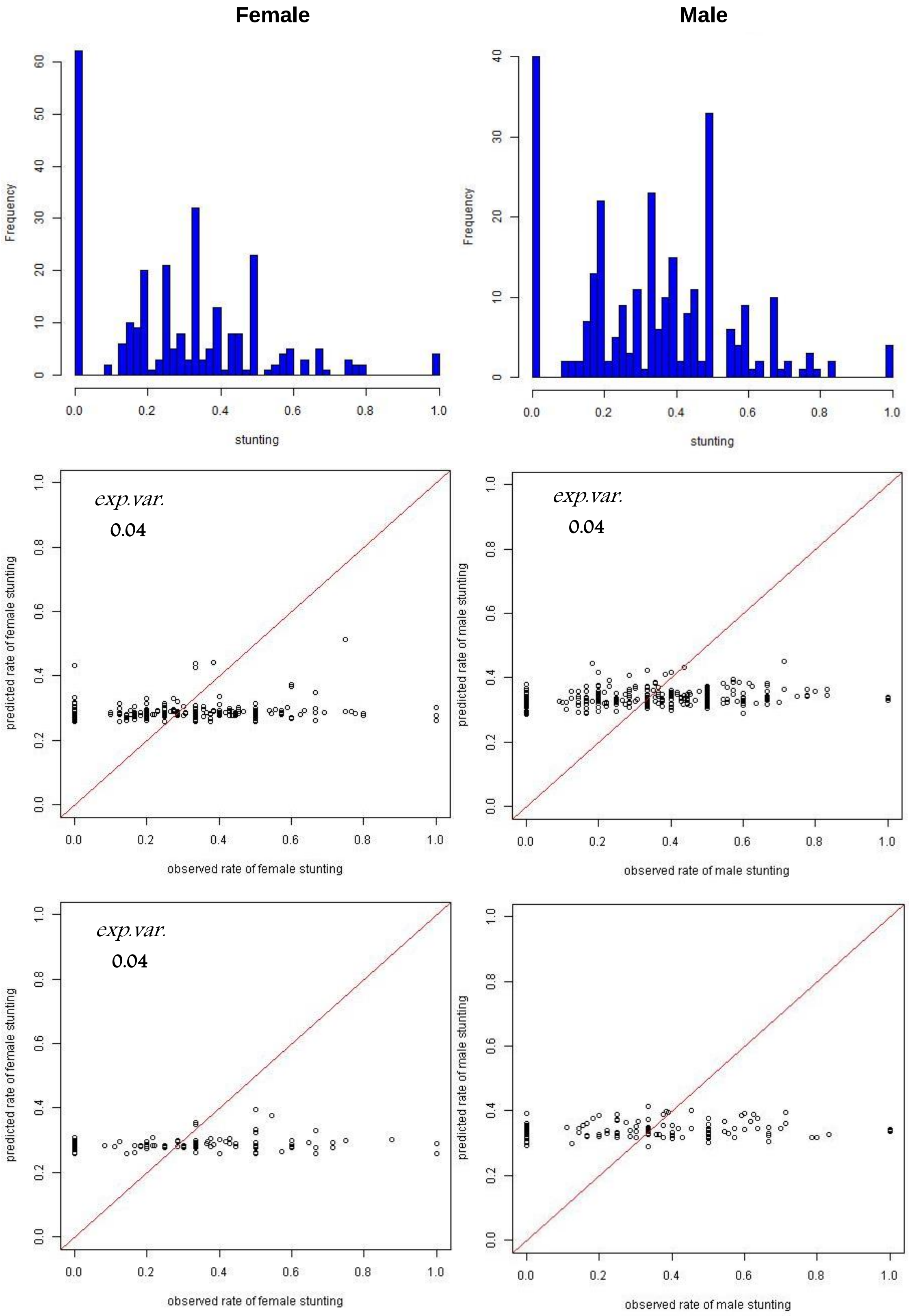

3.2.3 Bangladesh stunting

Figure 14. Gender disaggregated histograms (top row) related to the distribution of a 70% subset of DHS data (training) and scatter plot of the predicted (y-axis) and observed (x-axis) proportion of stunting for children under the age of 5, in the training (middle row) and validation (bottom row).

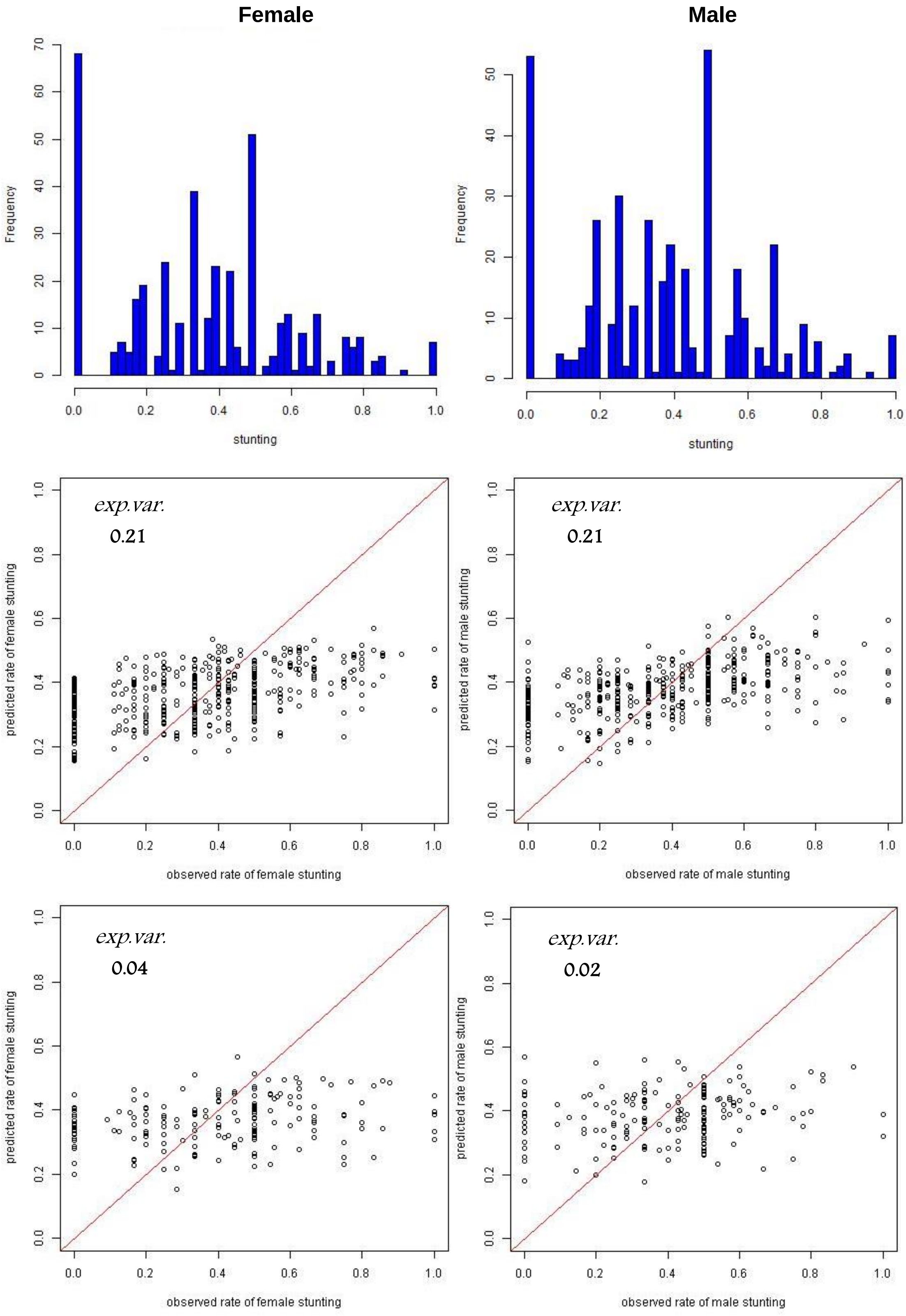

3.2.4 Stunting Covariate Selection

Table 6. Summary output from the covariate selection procedure for the indicator on male and female stunting in children under the age of 5 in Nigeria, Kenya, and Bangladesh. The *Exp. Var.* is the proportion of variance explained by the models.

Nigeria	Female	Male
**N. of covariates**	11	9
**Exp. Var.**	0.61	0.60
**Selected Covariates**	Distance to conflicts<br>Elevation<br>MID Infrared Index<br>Gross cell production<br>Percentage of Urban areas<br>Distance to roads<br>Distance to waterways<br>Pigs<br>Accessibility<br>Temperature<br>Rainfed crop suitability	Distance to conflicts<br>Elevation<br>MID Infrared Index<br>Gross cell production<br>Percentage of Urban areas<br>Distance to roads<br>Distance to waterways<br>Nightlight<br>Land cover

Kenya	Female	Male
**N. of covariates**	3	6
**Exp. Var.**	0.04	0.02
**Selected Covariates**	Accessibility<br>Distance to roads<br>Settlement distance	Accessibility<br>Distance to roads<br>Settlement distance<br>MID Infrared Index<br>Potential evapotranspiration<br>Elevation

Bangladesh	Female	Male
**N. of covariates**	11	12
**Exp. Var.**	0.04	0.02
**Selected Covariates**	Accessibility<br>Elevation<br>Percentage of Urban areas<br>Distance to roads<br>Distance to waterways<br>MODIS EVI	Accessibility<br>Elevation<br>Percentage of Urban areas<br>Distance to roads<br>Distance to waterways<br>MODIS EVI

	Potential Evapotranspiration<br>Aridity index<br>Chickens<br>Goats<br>Population	Potential Evapotranspiration<br>Temperature<br>Latitude<br>Longitude<br>Precipitation<br>Nightlight

3.3 Use of modern contraception methods

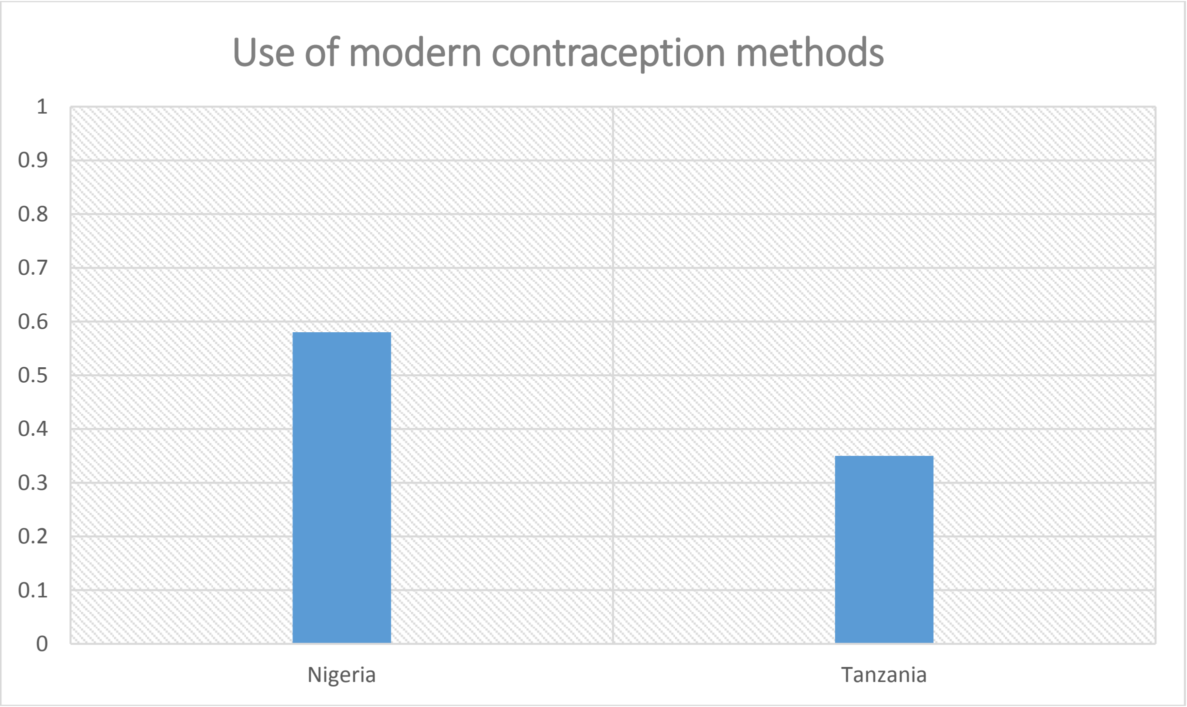

Figure 15. Explained variance of the maps related to the use of modern contraception methods in Nigeria and Tanzania.

3.3.1 Nigeria contraception

Figure 16a. Histogram (top left) related to the distribution of a 60% subset of DHS data (training) and scatter plot of the predicted (y-axis) and observed (x-axis) proportion of women aged 15-49 using modern contraception methods in the training (top right) and validation (bottom row) dataset.

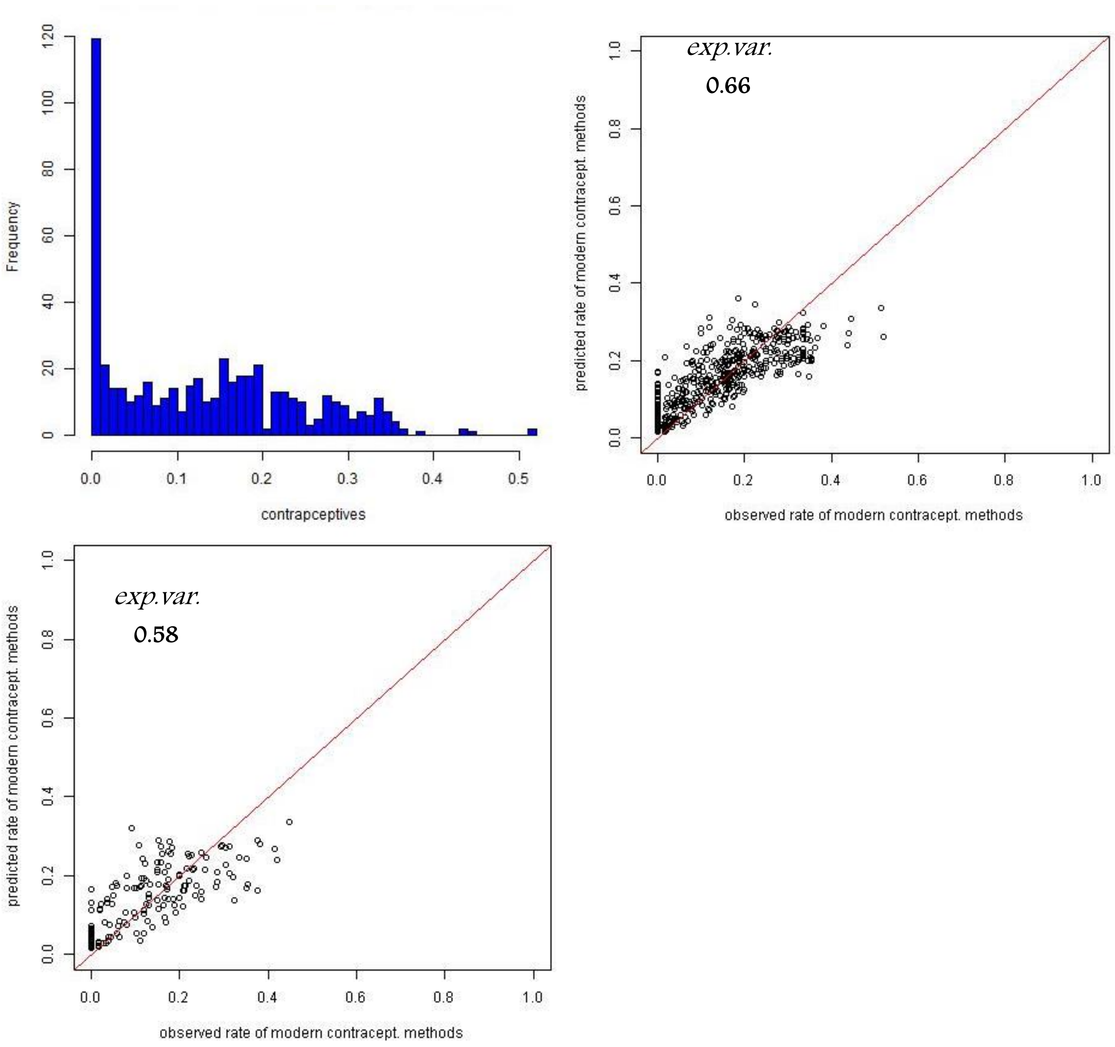

Figure 16b. Q-Q plot of the predicted (y-axis) and observed (x-axis) proportion of women using modern contraception methods in the training (left) and validation (right) dataset.

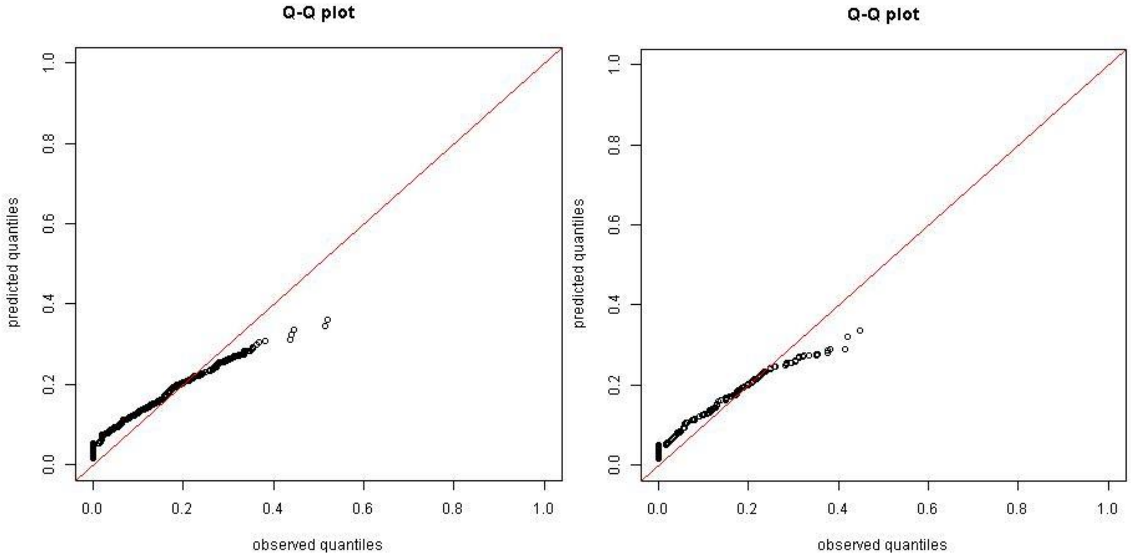

Figure 17. Map of the cluster-level survey data (top row), for the indicator on use of modern contraception methods in female age 15-49. Maps of the mean predicted proportion of women using modern contraception methods at 1 km^2 resolution (middle row) and related uncertainty maps (bottom row) showing the associated standard deviation.

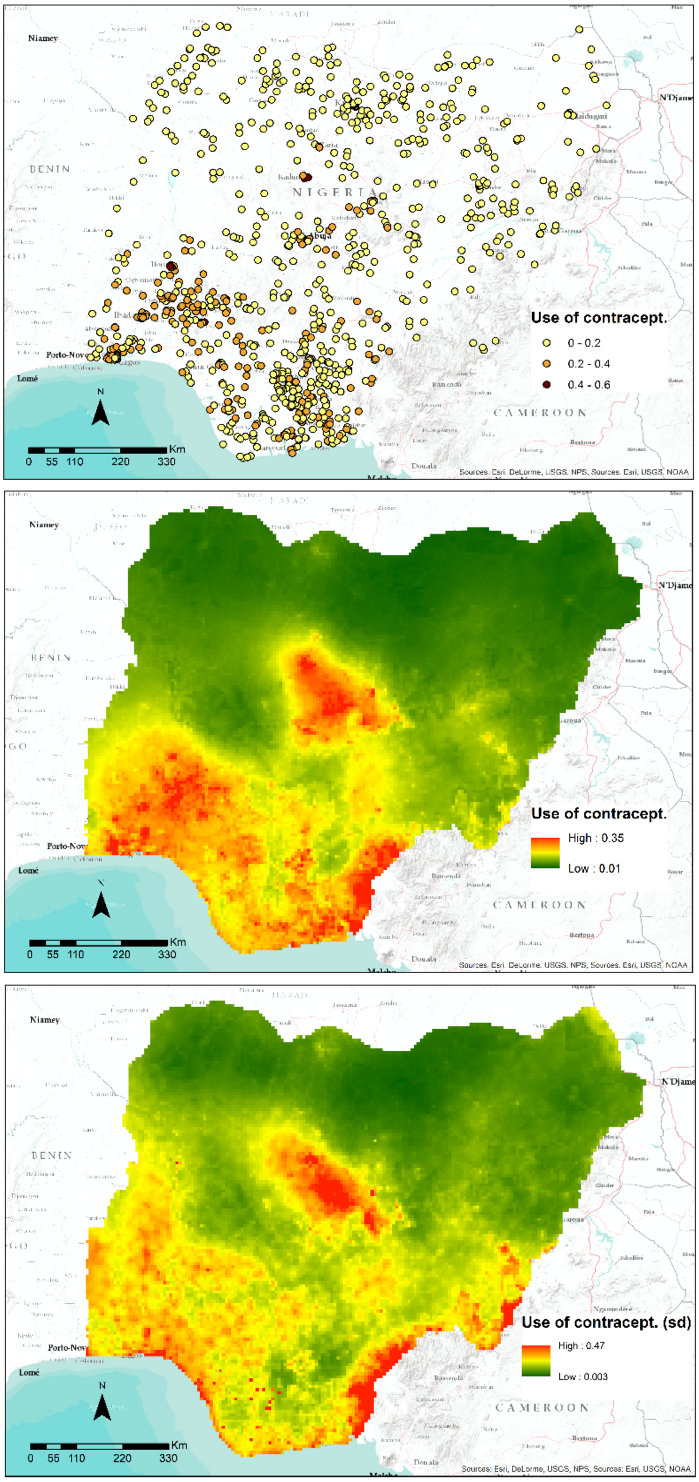

3.3.2 Tanzania contraception

Figure 18a. Histogram (top left) related to the distribution of the proportion of women aged 15-49 using modern contraception methods in a 70% subset of DHS data (training) and scatter plot of the predicted (y-axis) vs observed (x-axis) values in the training (top right) and validation (bottom row) dataset.

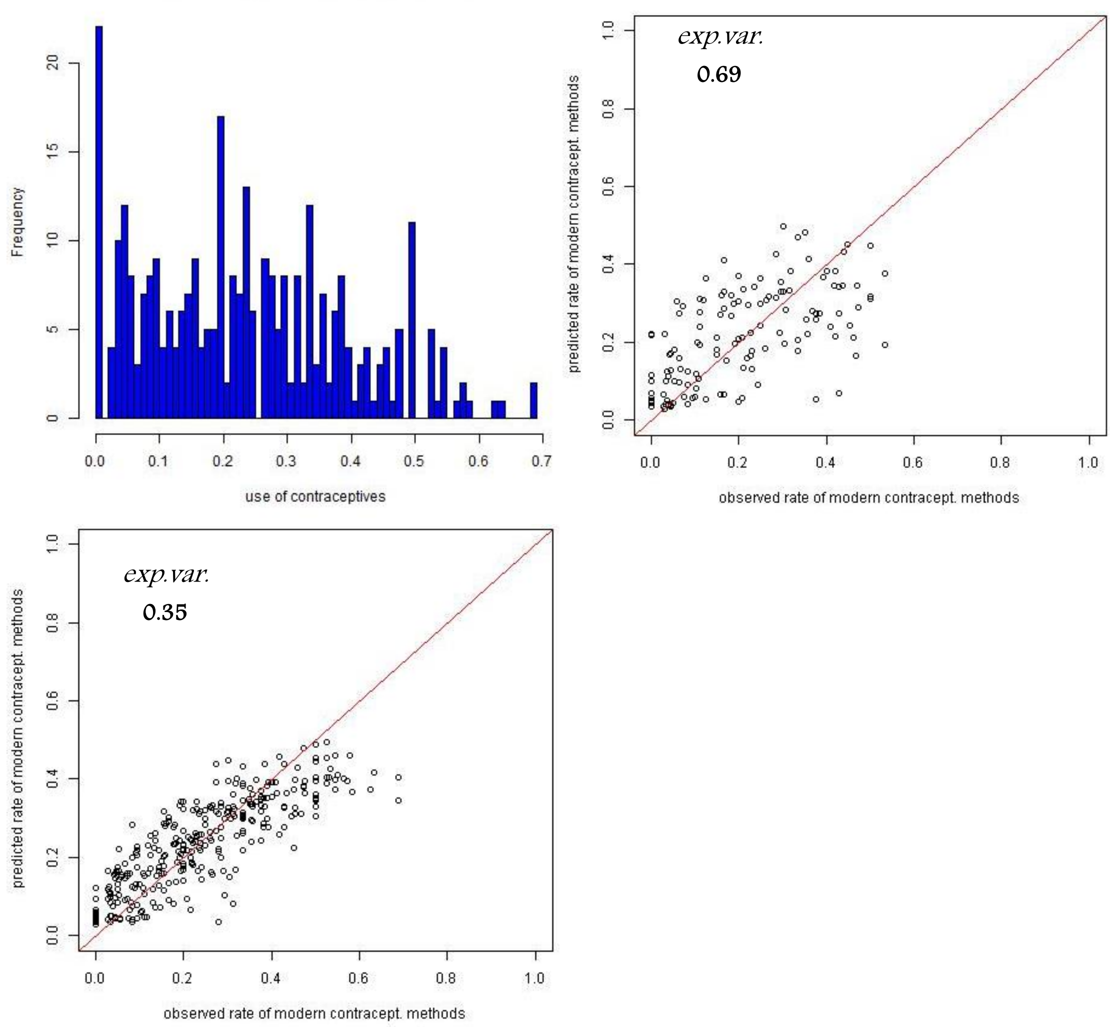

Figure 18b. Q-Q plot of the predicted (y-axis) and observed (x-axis) proportion of women using modern contraception methods in the training (left) and validation (right) dataset.

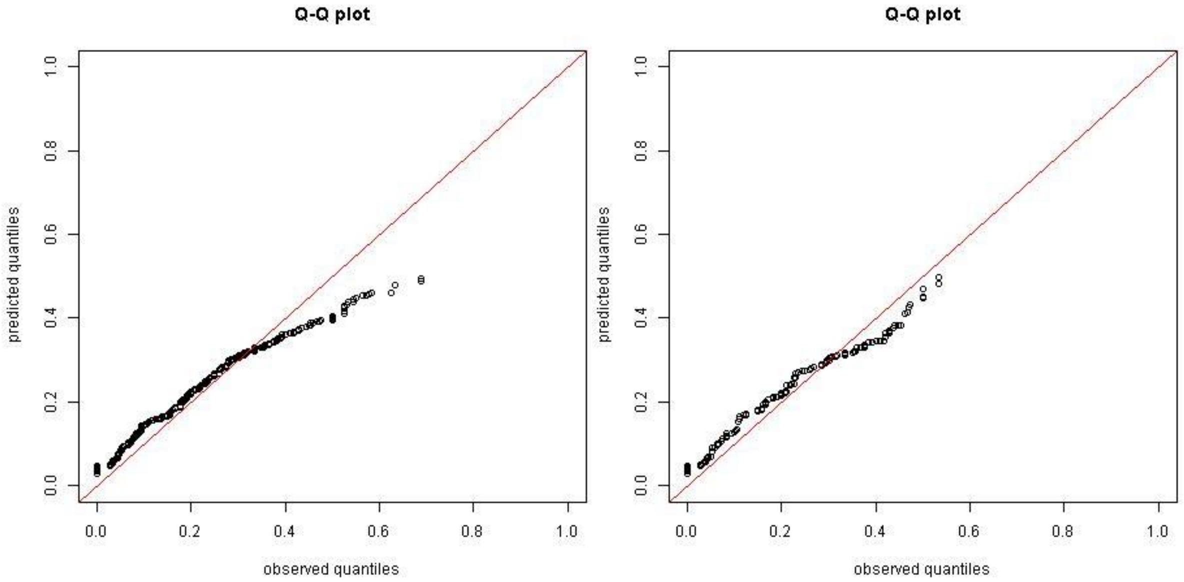

Figure 19. Map of the cluster-level survey data (top row), for the indicator on use of modern contraception methods in females age 15-49. Maps of the mean predicted proportion of women using modern contraception methods at 1 km^2 resolution (middle row) and related uncertainty maps (bottom row) showing the associated standard deviation.

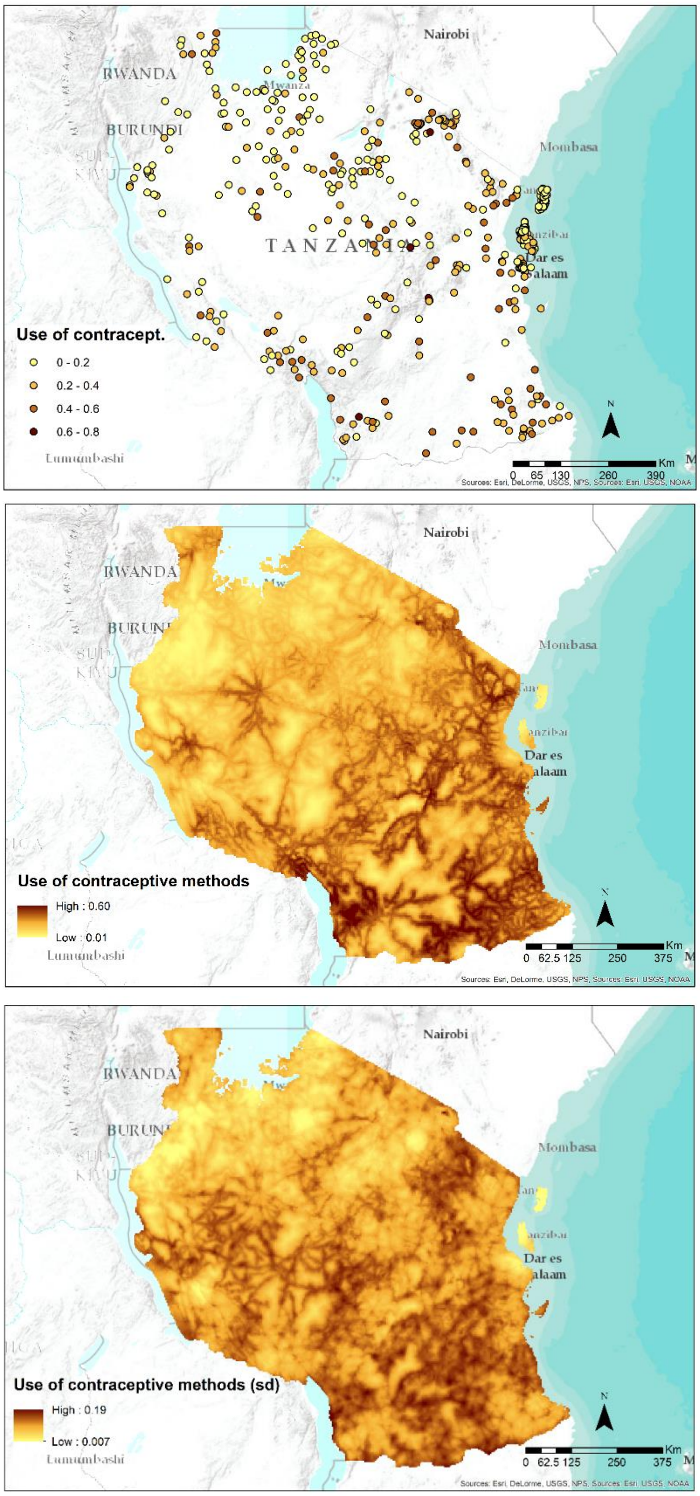

3.3.4 Contraception Covariate Selection

Table 7. Summary output from the covariate selection procedure for the indicator on the use of modern contraception methods for females aged 15-49 in Nigeria, Tanzania and Haiti. The *Exp. Var.* is the proportion of variance explained by the models.

	**Nigeria**
**N. of covariates**	11
**Exp. Var.**	0.58
**Selected Covariates**	Accessibility<br>Elevation<br>Cattle<br>Gross cell production<br>MODIS EVI<br>Nightlight<br>Percentage of urban areas<br>Potential evapotranspiration<br>Distance to roads<br>Distance to waterways<br>Rainfed crop suitability

	**Tanzania**
**N. of covariates**	9
**Exp. Var.**	0.35
**Selected Covariates**	Aridity index<br>Settlement distance<br>Distance to roads<br>MODIS EVI<br>Elevation<br>Accessibility<br>MID Infrared Index<br>Precipitation<br>Population

4 References

Alegana, V. A., P. M. Atkinson, C. Pezzulo, A. Sorichetta, D. Weiss, T. Bird, E. Erbach-Schoenberg, and A. J. Tatem (2015). "Fine resolution mapping of population age-structures for health and development applications." Journal of The Royal Society Interface 12, no. 105: 20150073.

Bosco, C., de Rigo, D., Dijkstra, T. A., Sander, G., Wasowski, J., 2013. Multi-scale robust modelling of landslide susceptibility: regional rapid assessment and catchment robust fuzzy ensemble. IFIP Advances in Information and Communication Technology 413, 321-335, ISSN:1868-4238. doi:10.1007/978-3-642-41151-9_31

Burgert, C.R., J. Colston, T. Roy, and B. Zachary. 2013. “Geographic Displacement Procedure and Georeferenced Data Release Policy for the Demographic and Health Surveys” DHS Spatial Analysis Reports No. 7 Calverton, Maryland, USA: ICF International.

Cameletti, M., Lindgren, F., Simpson, D., & Rue, H. (2013). Spatio-temporal modeling of particulate matter concentration through the SPDE approach. AStA Advances in Statistical Analysis, 97(2), 109-131.

Castejón Limas M., Ordieres Meré J.B., González Marcos A., Martínez de Pisón Ascacibar F.J., Pernía Espinoza A.V., Alba Elías F. : AMORE: A MORE Flexible Neural Network Package. R package version 0.2-12 2010 [http://www.inside-r.org/packages/amore/versions/0-2-12]

Cayemittes, Michel, Michelle Fatuma Busangu, Jean de Dieu Bizimana, Bernard Barrère, Blaise Sévère, Viviane Cayemittes et Emmanuel Charles. 2013. Enquête Mortalité, Morbidité et Utilisation des Services, Haïti, 2012. Calverton, Maryland, USA : MSPP, IHE et ICF International.

Center for International Earth Science Information Network – CIESIN. 2011 - Columbia University, International Food Policy Research Institute - IFPRI & The World Bank, and Centro Internacional de Agricultura Tropical - CIAT. Global Rural-Urban Mapping Project, Version 1 (GRUMPv1): Urban Extents Grid (Africa) at <http://sedac.ciesin.columbia.edu/data/set/grump-v1-urban-extents/maps>

de Rigo, D., Rizzoli, A. E., Soncini-Sessa, R., Weber, E., Zenesi, P., Dec. 2001. Neuro-dynamic programming for the efficient management of reservoir networks. In: Proceedings of MODSIM 2001, International Congress on Modelling and Simulation. Vol. 4. Modelling and Simulation Society of Australia and New Zealand, pp. 1949-1954. doi:10.5281/ZENODO.7481

de Rigo, D., 2012. Semantic Array Programming for environmental modelling: application of the Mastrave library. In: Seppelt, R., Voinov, A. A., Lange, S., Bankamp, D. (Eds.), International

Environmental Modelling and Software Society (iEMSs) 2012 International Congress on Environmental Modelling and Software. Managing Resources of a Limited Planet: Pathways and Visions under Uncertainty, Sixth Biennial Meeting. pp. 1167-1176. http://www.iemss.org/iemss2012/proceedings/D3_1_0715_deRigo.pdf

de Rigo, D., 2015. Study of a collaborative repository of semantic metadata and models for regional environmental datasets' multivariate transformations. Ph.D. thesis, Politecnico di Milano, Milano, Italy. INRMM-MiD:13769492

Eaton, J. W., Bateman, D., and Hauberg, S.: GNU Octave Manual Version 3. A high-level interactive language for numerical computations, Network Theory Limited, ISBN: 0-9546120-6-X, 2008.

Friedl, M. A., Sulla-Menashe, D., Tan, B., Schneider, A., Ramankutty, N., Sibley, A., andHuang, X. (2010). MODIS Collection 5 global land cover: Algorithm refinements and characterization of new datasets. Remote Sensing of Environment, 114, 168–182.

Gething, Peter, Andy Tatem, Tom Bird, and Clara R. Burgert-Brucker. 2015. Creating Spatial Interpolation Surfaces with DHS Data DHS Spatial Analysis Reports No. 11. Rockville, Maryland, USA: ICF International.

Ghassemieh, M., & Nasseri, M. (2012). Evaluation of Stiffened End-Plate Moment Connection through Optimized Artificial Neural Network. Journal of Software Engineering and Applications, 5(3), 156.

Hijmans, R. J., Cameron, S. E., Parra, J. L., Jones, P. G. & Jarvis, A. Very high resolution interpolated climate surfaces for global land areas. Int. J. Climatol. 25, 1965–1978 (2005).

Hornik, K., Stinchcombe, M., White, H., 1989. Multilayer feedforward networks are universal approximators. Neural Networks 2 (5), 359-366. doi:10.1016/0893-6080(89)90020-8

ICF International. (2012). Demographic and Health Survey Sampling and Household Listing Manual: MEASURE DHS, Calverton, Maryland, U.S.A.: ICF International.

Iverson, K. E., 1980. Notation as a tool of thought. Communications of the ACM 23 (8), 444-465. doi:10.1145/358896.358899.

Kenya National Bureau of Statistics (KNBS) and ICF Macro. 2010. Kenya Demographic and Health Survey 2008-09. Calverton, Maryland: KNBS and ICF Macro.

Kreinovich, V. Y., 1991. Arbitrary nonlinearity is sufficient to represent all functions by neural networks: a theorem. Neural Networks 4 (3), 381-383. doi:10.1016/0893-6080(91)90074-f

Lee, S., Ryu, J.H., Kim, I.S.: Landslide susceptibility analysis and its verification using likelihood ratio, logistic regression, and artificial neural network models: case study of Youngin, Korea. Landslide 4, (2007) 327-338

Macro International Inc. 1996. Sampling manual: DHS III basic documentation. Carlverton, MD: Macro International Inc

National Bureau of Statistics (NBS) [Tanzania] and ICF Macro. 2011.
Tanzania Demographic and Health Survey 2010. Dar es Salaam, Tanzania: NBS and ICF Macro.

National Institute of Population Research and Training (NIPORT), Mitra and Associates, and ICF
International. 2013. Bangladesh Demographic and Health Survey 2011. Dhaka, Bangladesh and
Calverton, Maryland, USA: NIPORT, Mitra and Associates, and ICF International.

National Population Commission (NPC) [Nigeria] and ICF International. 2014. Nigeria Demographic and
Health Survey 2013. Abuja, Nigeria, and Rockville, Maryland, USA: NPC and ICF International.

Nordhaus, W., Azam, Q., Corderi, D., Hood, K., Victor, N. M., Mohammed, M., ... & Weiss, J. (2006). The G-Econ database on gridded output: methods and data. Yale University, New Haven.

Perez-Heydrich, Carolina, J. L. W., Clara R. Burgert and Emch, M. E. (2013). Guidelines on the Use of DHS GPS Data. Spatial Analysis Reports No. 8. Calverton, Maryland, USA: ICF International.

Pradhan, B., Lee, S.: Landslide risk analysis using artificial neural network model focusing on different training sites. International Journal of Physicical Sciences 4(1), (2009) 1-15

Quenouille, M. H. (September 1949). "Problems in Plane Sampling". The Annals of Mathematical Statistics 20 (3): 355–375. doi:10.1214/aoms/1177729989. JSTOR 2236533.

Quenouille, M. H. (1956). "Notes on Bias in Estimation". Biometrika 43 (3-4): 353–360. doi:10.1093/biomet/43.3-4.353. JSTOR 2332914.

Robinson, T. et al. Mapping the Global Distribution of Livestock. PLos ONE 9, (2014).

Rosenblatt, F., 1962. Principles of Neurodynamics: Perceptrons and the Theory of Brain Mechanisms, Spartan, Washington DC.

Rutstein, S. and Rojas, G. (2003). Guide to DHS Statistics: Demographic and Health Surveys, ORC Macro, Calverton, Maryland.

Schmid M.D., 2009: A neural network package for Octave. User's Guide Version: 0.1.9.1. http://www.plexso.com/61_octave/neuralNetworkPackageForOctaveUsersGu.pdf

Secomandi, N., 2000. Comparing neuro-dynamic programming algorithms for the vehicle routing problem with stochastic demands. Computers & Operations Research 27 (11–12): 1201–1225. doi:10.1016/S0305-0548(99)00146-X

Sedda, L., Tatem, A. J., Morley, D. W., Atkinson, P. M., Wardrop, N. A., Pezzulo, C., ... & Rogers, D. J. (2015). Poverty, health and satellite-derived vegetation indices: their inter-spatial relationship in West Africa. International health, 7(2), 99-106.

Tatem, A. J., Noor, A. M., & Hay, S. I. (2004). Defining approaches to settlement mapping for public health management in Kenya using medium spatial resolution satellite imagery. Remote Sensing of Environment, 93(1), 42-52.

Tatem, A. J., Noor, A. M., & Hay, S. I. (2005). Assessing the accuracy of satellite derived global and national urban maps in Kenya. Remote sensing of environment, 96(1), 87-97.

Taylor, S., 2003. Extreme terseness: Some languages are more agile than others. Lecture Notes in Computer Science 2675, 334-336. doi:10.1007/3-540-44870-5_44.

Thome, K. Terra | The EOS Flagship. at <http://terra.nasa.gov/>

Tukey, J. W. (1958). "Bias and confidence in not quite large samples". The Annals of Mathematical Statistics 29: 614–623. doi:10.1214/aoms/1177706647.

Venables, W.N. and B. D. Ripley: "Modern Applied Statistics with S.", Fourth edition, Springer, 2002.

Vogt, M. et al. Integrating Data on Ethnicity, Geography, and Conflict The Ethnic Power Relations Data Set Family. J. Confl. Resolut. 59, 1327–1342 (2015).

5 Code

5.1 Code in GNU Octave

```
%   Author: Claudio Bosco
%   This code provides a general example that exploits the package
%   NNET (GNU Octave) to train and validate a neural network in order
%   to predict gender disaggregated development indicators from the
%   combination of geolocated Demographic and Health Surveys (DHS)
%   cluster data and geospatial covariates.
%   Copyright (C) 2015,2016,2017 University of Southampton
%
%   This program is free software: you can redistribute it and/or modify
%   it under the terms of the GNU General Public License as published by
%   the Free Software Foundation, either version 3 of the License, or
%   (at your option) any later version.
%
%   This program is distributed in the hope that it will be useful,
%   but WITHOUT ANY WARRANTY; without even the implied warranty of
%   MERCHANTABILITY or FITNESS FOR A PARTICULAR PURPOSE.  See the
%   GNU General Public License for more details.
%
%   You should have received a copy of the GNU General Public License
%   along with this program.  If not, see <http://www.gnu.org/licenses/>.
%
%
% Dependencies:
%    Package     Version               URL
%    __________  ____________________  _____________________________________
%    GNU Octave  3.2.x or successive   https://gnu.org/software/octave/
%    Mastrave    0.2.x or successive   http://mastrave.org
%    nnet        0.1.13 or successive  https://octave.sourceforge.io/nnet/
%
% The array-based semantic checks as defined in the Semantic Array Programming (SemAP)
% paradigm are described at: http://mastrave.org/doc/mtv_m/check_is
%
%
%
% This script requires the following variables to be already defined
%    <data_valid>     : this ::matrix:: is the dataset used for validation
%    <data_train>     : this ::matrix:: is the dataset used for training
%    <depend_var_pos> : this ::scalar:: ::index:: is the column position of the
%                       dependent variable within <data_valid> and <data_train>
%    <covar_pos>      : this ::row_vector:: ::index:: is the set of column positions
%                       of the selected covariates within <data_valid> and <data_train>
%    <nHiddenNeurons> : this variable is the number of hidden neurons
%    <cvrun>          : this is the number of run of cross-validation
%    <num>            : this is the number of run of the ANN
%



%-----------------------------------------------------------------------%
%                      Feed-forward backprop network                    %
%-----------------------------------------------------------------------%

where           = sprintf(                                                ...
   gettext( '(in the script %s)' )                                      , ...
```

```
   mfilename                                                                    ...
);

nrun                         = size( data_train, 1 );
[ nRows_val , nColumns_val ] = size( data_valid );



% set training and validation output
mOutput      = data_train(:,depend_var_pos); % extract dependant variable in training
mValidOutput = data_valid(:,depend_var_pos); % extract dep. variab. in validation



check_is(   mOutput ,     'col_vector'                                        , ...
   gettext( [                                                                   ...
      '%s the variable <%s> must be a column vector'                            ...
   ] )                                                                        , ...
   where, 'mOutput'                                                             ...
);


check_is(   mValidOutput , 'col_vector'                                       , ...
   gettext( [                                                                   ...
      '%s the variable <%s> must be a column vector'                            ...
   ] )                                                                        , ...
   where, 'mValidOutput'                                                        ...
);


check_is(   mOutput ,      'real'                                             , ...
   gettext( [                                                                   ...
      '%s the variable <%s> must be of real values'                             ...
   ] )                                                                        , ...
   where, 'mOutput'                                                             ...
);


check_is(   mValidOutput , 'real'                                             , ...
   gettext( [                                                                   ...
      '%s the variable <%s> must be of real values'                             ...
   ] )                                                                        , ...
   where, 'mValidOutput'                                                        ...
);


Input     = data_train( :, covar_pos );
Input_val = data_valid( :, covar_pos );


check_is(   Input ,    'matrix'                                               , ...
   gettext( [                                                                   ...
      '%s the variable <%s> must be a matrix'                                   ...
   ] )                                                                        , ...
   where, 'Input'                                                               ...
);
```

```
check_is(   Input_val , 'matrix'                                         , ...
   gettext( [                                                              ...
      '%s the variable <%s> must be a matrix'                              ...
   ] )                                                                   , ...
   where, 'Input_val'                                                      ...
);


check_is(   Input ,    'real'                                            , ...
   gettext( [                                                              ...
      '%s the variable <%s> must be of real values'                        ...
   ] )                                                                   , ...
   where, 'Input'                                                          ...
);


check_is(   Input_val , 'real'                                           , ...
   gettext( [                                                              ...
      '%s the variable <%s> must be of real values'                        ...
   ] )                                                                   , ...
   where, 'Input_val'                                                      ...
);


check_is(   { mOutput,      Input     }, 'same_rows'                     , ...
   gettext( [                                                              ...
      '%s the variables <%s> and <%s> must have the same number of rows'   ...
   ] )                                                                   , ...
   where, 'mOutput', 'Input'                                           ...
);

check_is(   { mValidOutput, Input_val }, 'same_rows'                     , ...
   gettext( [                                                              ...
      '%s the variables <%s> and <%s> must have the same number of rows'   ...
   ] )                                                                   , ...
   where, 'mValidOutput', 'Input_val'                                      ...
);


N_train_data = size( mOutput,      1 )
N_valid_data = size( mValidOutput, 1 )


% transposed of the initial training and validation dataset
mInput       = Input';
trainOutput  = mOutput';
% check_is( { trainOutput,  mInput    }, 'same_columns' );
check_is(   { trainOutput,  mInput    }, 'same_columns'                    , ...
   gettext( [                                                              ...
      '%s the variables <%s> and <%s> must have the same number of columns'   ...
   ] )                                                                   , ...
   where, 'trainOutput', 'mInput'                                          ...
);

% set validation data
% transposed
mValidInput  = Input_val';
validOutput  = mValidOutput';
```

```
% check_is( { mValidInput, validOutput }, 'same_columns' );
check_is(   { mValidInput, validOutput }, 'same_columns'                    , ...
   gettext( [                                                              ...
      '%s the variables <%s> and <%s> must have the same number of columns'   ...
   ] )                                                                   , ...
   where, 'mValidInput', 'validOutput'                                     ...
);

% standardize training inputs
meanInput    = mean( mInput,     2 ); % calculate the mean of each row
sInput       = std(  mInput, [], 2 );
trainInput   = bsxfun( @rdivide, bsxfun( @minus, mInput,      meanInput ), sInput );

% standardize validation inputs
validInput   = bsxfun( @rdivide, bsxfun( @minus, mValidInput, meanInput ), sInput );



% Function for performing the cross-validation
% <train_id>   ::index::
function [ ttrain, tvalid ] = crosstrainvalid( train_id )

   n        = numel( train_id );
   id       = randperm( n );
   ttlid    = linspace( 0, 100, n ) < 70;
   train_id = train_id( id );
   ttrain   = train_id(  ttlid );
   tvalid   = train_id( ~ttlid );

end



% network training

nOutputNeurons = 1;


for run = 1:cvrun
   [ttrain,tvalid] = crosstrainvalid( 1:nrun );


   tt_out = mOutput( ttrain, : );
   tt_in  = Input(   ttrain, : );
   tv_out = mOutput( tvalid, : );
   tv_in  = Input(   tvalid, : );
   msetot = [];

   [nRows_t, nColumns]     = size( tt_in );
   [nRows_v, nColumns_val] = size( tv_in );

   % transposed of the crossvalid training and validation
   tt_in  = tt_in.';
   tt_out = tt_out';


   % set validation data
   % transposed
```

```
   tv_in  = tv_in.';
   tv_out = tv_out';

   % standardize training inputs
   meanInput   = mean( tt_in, 2 ); #calculate the mean of each row
   sInput      = std( tt_in, [], 2 );
   trainInput  = bsxfun( @rdivide, bsxfun( @minus, tt_in, meanInput ), sInput );

   % standardize validation inputs
   tvalidInput = bsxfun( @rdivide, bsxfun( @minus, tv_in, meanInput ), sInput );

   MinMaxElements = min_max(trainInput);

   for j = 1:num

      MLPnet = newff(
         MinMaxElements                  ,
         [nHiddenNeurons nOutputNeurons] ,
         {"tansig","purelin"}            ,
         "trainlm"                       ,
         "learngdm"                      ,
         "mse"
      );
      net.trainParam.epochs   = 300;
      net.trainParam.max_fail = 5;
      net.trainParam.goal     = 0.05;

      net_training{j} = train(MLPnet, trainInput, tt_out);
      y_train         = sim(net_training{j}, trainInput);
      MSE_train       = (sum((tt_out - y_train).^2))/nRows_t; % MSE crossvalid training

      y_valid         = sim(net_training{j}, tvalidInput);
      MSE_valid       = (sum((tv_out - y_valid).^2))/nRows_v; % MSE crossvalid validation
      msetot(j)       = MSE_train + MSE_valid;

   end

   [~, best_j ]      = min(msetot);
   selected_net{run} = net_training{best_j};

end

% ...
```

GNU–Octave_SemAP_code.m

5.2 Code in GNU R

```
#   Author: Claudio Bosco
#   This code provides a general example that exploits the package
#   AMORE (GNU R) to train and validate a neural network in order to
#   predict gender disaggregated development indicators from the
#   combination of geolocated Demographic and Health Surveys (DHS)
#   cluster data and geospatial covariates.
#   Copyright (C) 2015,2016,2017 University of Southampton
#
#   This program is free software: you can redistribute it and/or modify
#   it under the terms of the GNU General Public License as published by
#   the Free Software Foundation, either version 3 of the License, or
#   (at your option) any later version.
#
#   This program is distributed in the hope that it will be useful,
#   but WITHOUT ANY WARRANTY; without even the implied warranty of
#   MERCHANTABILITY or FITNESS FOR A PARTICULAR PURPOSE.  See the
#   GNU General Public License for more details.
#
#   You should have received a copy of the GNU General Public License
#   along with this program.  If not, see <http://www.gnu.org/licenses/>.
#
#
# Dependencies:
#    Package      Version               URL
#    ___________  ____________________  ______________________________________________
#    GNU R        3.1.x or successive   https://gnu.org/software/r/
#    AMORE        0.2-15 or successive  https://cran.r-project.org/package=AMORE
#    matrixStats  0.51.x or successive  https://cran.r-project.org/package=matrixStats

library( AMORE )
require( matrixStats )

#
#  This script requires the following variables to be already defined
#    <test>            : this ::matrix:: is the dataset used for the final test
#    <train>           : this ::matrix:: is the dataset used for training
#    <depend_var_pos> : this ::scalar:: ::index:: is the column position of the
#                        dependent variable within <data_valid> and <data_train>
#    <est>             : this ::scalar:: ::index:: is the number of cycles of
#                        crossvalidation
#    <num>             : this ::scalar:: ::index:: is the number of run of the loop

# Furthermore, the following predictor variables (used for modelling
# male literacy in Tanzania) are expected to be defined.
# In alternative, any other set of predictors might be used instead.
# In this case, please adapt the lines 33-40, 58-65, 69-76

# ...

test_final <- test[,c(
   "aridity_index"  ,
   "nightlight"     ,
   "roads_distance" ,
   "precipitation"  ,
   "MODIS_EVI"      ,
   "elevation"      ,
```

```
   "latnum"         ,
   "accessibility"
)]


est_valid = matrix(0,est,dim(test_final)[1])

for (run in 1:est) {

   loopcheck = Inf
   newff_index <- sample(
      1:nrow(train),
      size   = 0.3*nrow(train)
   )

   # Training set of data within the crossvalidation loop
   ttrain    = train[-newff_index,]
   tvalid    = train[newff_index,]

   var1      <- ttrain[,c(
      "aridity_index"  ,
      "nightlight"     ,
      "roads_distance" ,
      "precipitation"  ,
      "MODIS_EVI"      ,
      "elevation"      ,
      "latnum"         ,
      "accessibility"
   )]

   val       <- tvalid[,c(
      "aridity_index"  ,
      "nightlight"     ,
      "roads_distance" ,
      "precipitation"  ,
      "MODIS_EVI"      ,
      "elevation"      ,
      "latnum"         ,
      "accessibility"
   )]

   train_obs <- train[-newff_index,][,depend_var_pos]
   valid_obs <- train[ newff_index,][,depend_var_pos]


   # As an example we create a feedforward network, with two hidden layers both
   # with three neurons.
   # The hidden layers have got Tansig activation functions and the output
   # layer is Purelin.

   for (j in 1:num) {

      net    <- newff(
         n.neurons            = c(8,3,3,1), # Please, feel free to adapt this default ANN
         learning.rate.global = 0.01,
         momentum.global      = 0.5,
         error.criterium      = "LMS",
         Stao                 = NA,
```

```
         hidden.layer          = "tansig",
         output.layer          = "purelin",
         method                = "ADAPTgd"
      )
      # All the parameter values (e.g. learning rate, error. criterium, number of
      # neurons and hidden layers, etc.) need to be adjusted during the training by
      # analysing the output of the network.

      result <- train( net, var1, train_obs, report=TRUE, show.step=100, n.shows=5 )

      # Calculate SSE and MSE for crossvalid training
      predict.result.ann <- sim(result$net, var1)
      train.sse.ANN      <- sum((as.numeric(train_obs) - predict.result.ann)^2)
      train.MSE.ANN      <- train.sse.ANN /(dim(var1) [1]) # calculate MSE

      # Calculate SSE and MSE for crossvalid validation
      predict.validation.result.ANN <- sim(result$net, val)
      valid.sse.ANN      <- sum( ( as.numeric(valid_obs) -
                                   predict.validation.result.ANN )^2 )
      valid.MSE.ANN      <- valid.sse.ANN /(dim(val) [1]) # calculate MSE

      if((train.MSE.ANN + valid.MSE.ANN) < loopcheck) {
         loopcheck    = (train.MSE.ANN + valid.MSE.ANN)
         MSE.train    = train.MSE.ANN
         MSE.valid    = valid.MSE.ANN
         selected_net = result$net
      }

   }


est_valid[run,] = sim( selected_net, test_final )

}

# ...
# Following the population of <est_valid> with the estimates for each run, a simple
# robust aggregation may be constituted by their median.
```

GNU-R_code.R
